# Data for high-throughput screening of enzyme mutants by comparison of their activity ratios to an enzyme tag

**DOI:** 10.1016/j.dib.2019.104985

**Published:** 2019-12-13

**Authors:** Yaping Li, Huimin Chong, Xiang Zhang, Xiaolan Yang, Fei Liao

**Affiliations:** a School of Laboratory Medicine, Chongqing Medical University, Chongqing 400016, China; b School of Pharmacy and Bioengineering, Chongqing University of Technology, Chongqing 400054, China

**Keywords:** Mutant libraries, Enzyme tag, Fusion expression, Activity ratio, HTP screening

## Abstract

Data in this article are associated with the research article “High-throughput screening of enzyme mutants by comparison of their activity ratios to an enzyme tag” (Li et al., 2019) [1]. Data are provided on the development of a system for high-throughput (HTP) screening of mutants through the comparison of the activity ratios of an applicable enzyme and its mutants to a suitable tag enzyme in cell lysates of their fused forms, with *Escherichia coli* alkaline phosphatase (ECAP) as the tag fused to the *N*-terminus of *Pseudomonas Aeruginosa* arylsulfatase (PAAS) and its mutants *via* a flexible linker. Data were made publicly available for further analyses.

**Table:** Specifications Table

Subject	Chemistry, Biology
Specific subject area	Biomolecule engineering
Type of data	Figure, Table
How data were acquired	The adsorbance change for enzyme activity assay was recorded with Biotek ELX 800 at room temperature.
Data format	Raw and Analyzed
Parameters for data collection	The mixture of cell lysate of 20 μL and substrate of 180 μL containing both or one of final 2.0 mM 4NPS and final 0.20 mM 4NNPP in a well was agitated for 2.0 min at room temperature, to record the absorbance in 30 min after the total lagging time of 4.0 min.
Description of data collection	Fusion expression of ECAP with PAAS and its mutants in DE3; Protein bands after SDS-PAGE staining with Coomassie brilliant blue or antibodies following standard protocol; SDESA or separate single assay of ECAP and PAAS/mutant to derive their activity ratios.
Data source location	Chongqing Medical University, Chongqing 400016, China
Data accessibility	Data incorporated within this article
Related research article	Yaping Li, Huimin Chong, Xiang Zhang, Xiaolan Yang,*, Fei Liao* High-throughput screening of enzyme mutants by comparison of their activity ratios to an enzyme tag Analytical Biochemistry doi: 10.1016/j.ab.2019.113474

**Table:** 

**Value of the Data** • The dataset in this report will facilitate understanding the validation and application of a new high-throughput screening strategy to mutants of an enzyme in a library [1]. To recognize positive mutants in a library, this new screening strategy fuses enzyme/mutants with a tag enzyme to compare activity ratios of the enzyme/mutants to the tag enzyme in cell lysates of their fused forms, when such activity ratios have physical significance and are proportional to specific activities of the non-fused counterpart enzyme/mutants. • Re-analyses of these data will benefit researchers to develop a practical system of the new high-throughput screening strategy for directed evolution of an applicable enzyme. • The data in this report utilizes *Escherichia coli* alkaline phosphatase (ECAP) as the tag enzyme for fusion with *Pseudomonas Aeruginosa* arylsulfatase (PAAS) *via* a flexible linker. Through the analyses of the data of the activities of ECAP in lysates of both *Escherichia coli* BL21 (DE3) transformed with a blank plasmid and host cells transformed with the fused mutants of PAAS, a rational threshold of ECAP activities in cell lysates can be developed for physical significance of the activity ratios of their fused forms at a preset confidence limit. Meanwhile, with a focused library of PAAS mutants through saturated mutagenesis at M72, the data enable researchers to understand the proportionality between the activity ratios of PAAS/mutants to ECAP in cell lysates of their fused forms and specific activities of the non-fused counterpart PAAS/mutants. • The data in this report will provide insights on the application of the new screening strategy to the elucidation of sequence-activity relationship of an applicable enzyme.

## 1 Data description

**Fig. 1:**
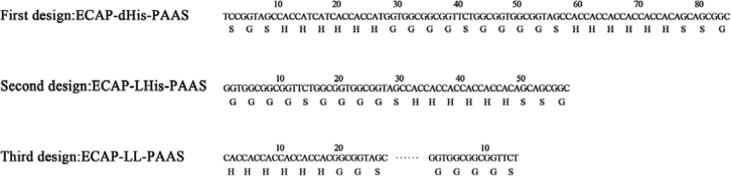
Three designed linkers for fusion expression of ECAP and PAAS.

**Fig. 2:**
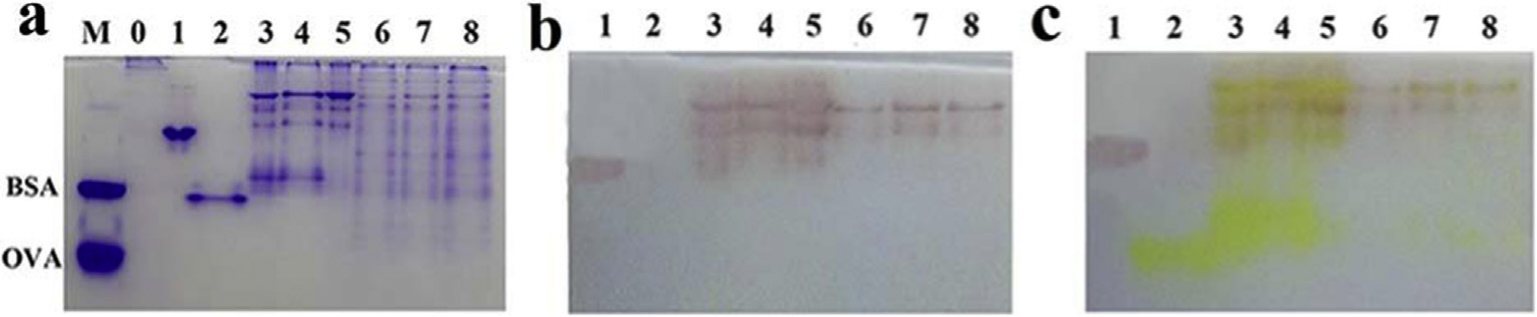
PAGE analyses of fragmentation patterns. Based on the Western blotting with polyclonal antibodies against PAAS, ECAP, monoclonal antibody against 6His tag, and Coomassie Blue R250 staining of polypeptides after SDS-PAGE in Fig. 1a, b, c, d in Ref. [1], respectively, here are the detection of polypeptides after separation by PAGE. (a) Coomassie Blue R250 staining. (b) ECAP activity staining with 1-Naphthol phosphate. (c) PAAS activity staining with 4-Nitrophenylsulfate on the same gel used in (b) after staining of ECAP activities.

**Fig. 3:**
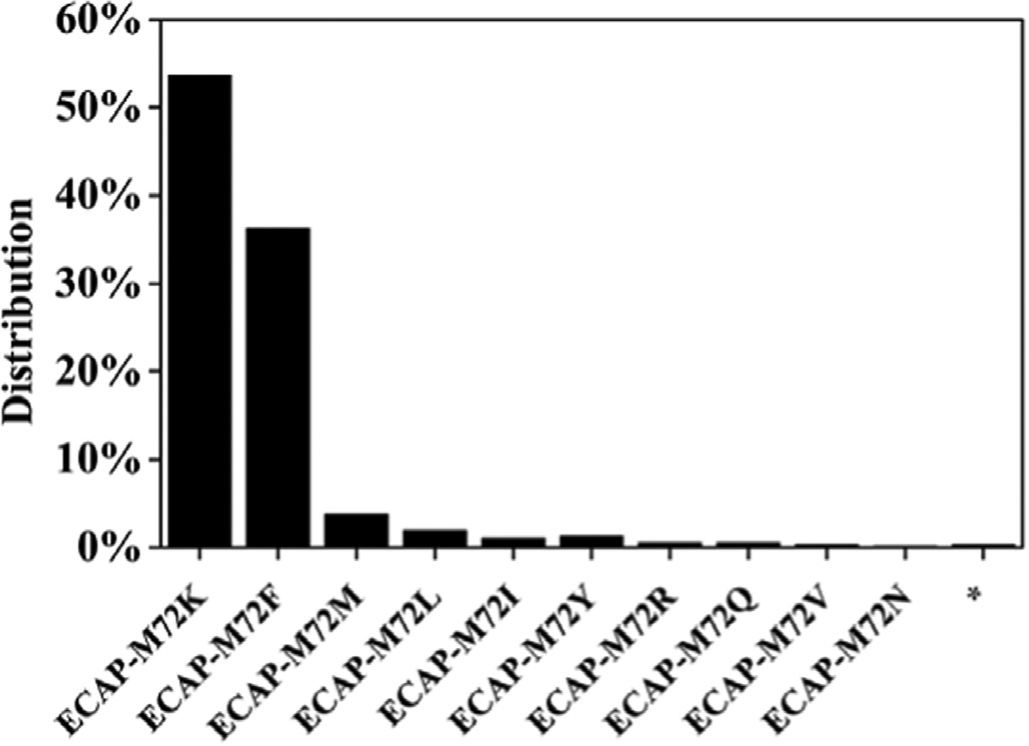
Distributions of PAAS mutants after saturation mutagenesis at M72. * represents the three termination codes.

**Fig. 4:**
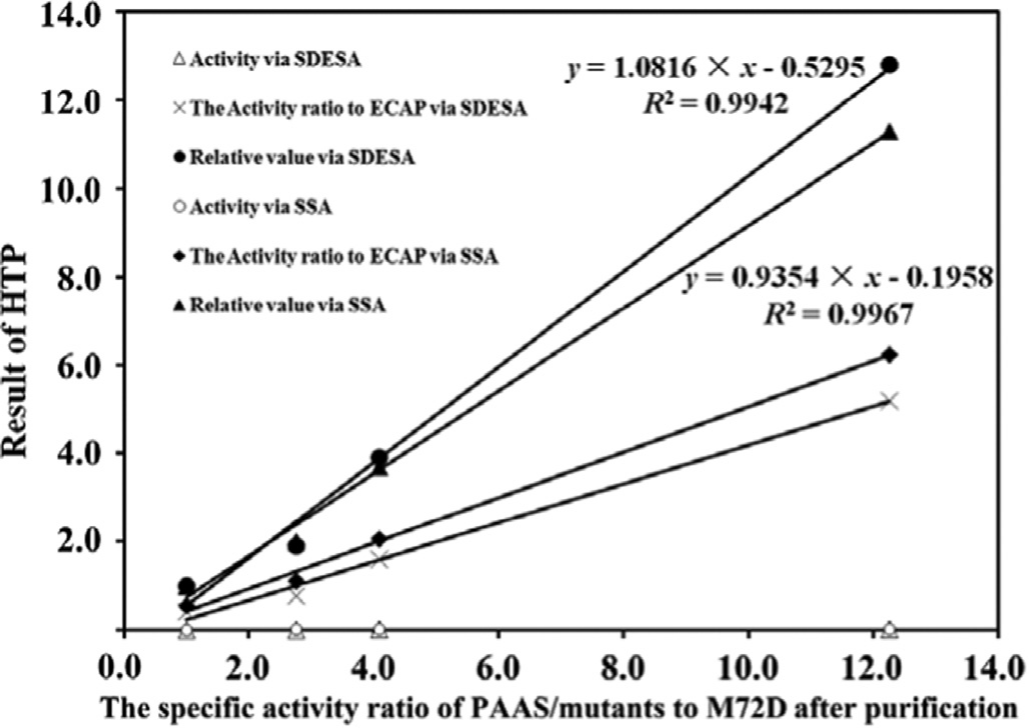
Association of activity ratios of the fused forms in cell lysates with specific activities of their purified non-fused counterparts.

**Fig. 5:**
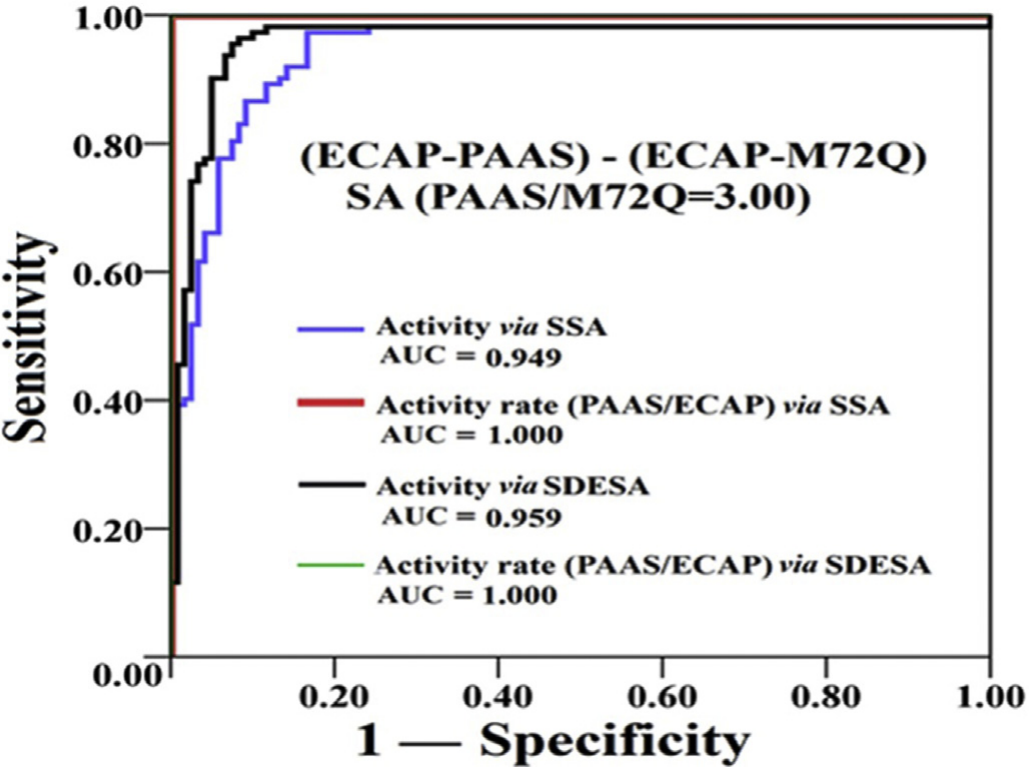
ROC analyses of the recognition of hits in fused PAAS versus fused M72Q.

**Scheme. 1:**
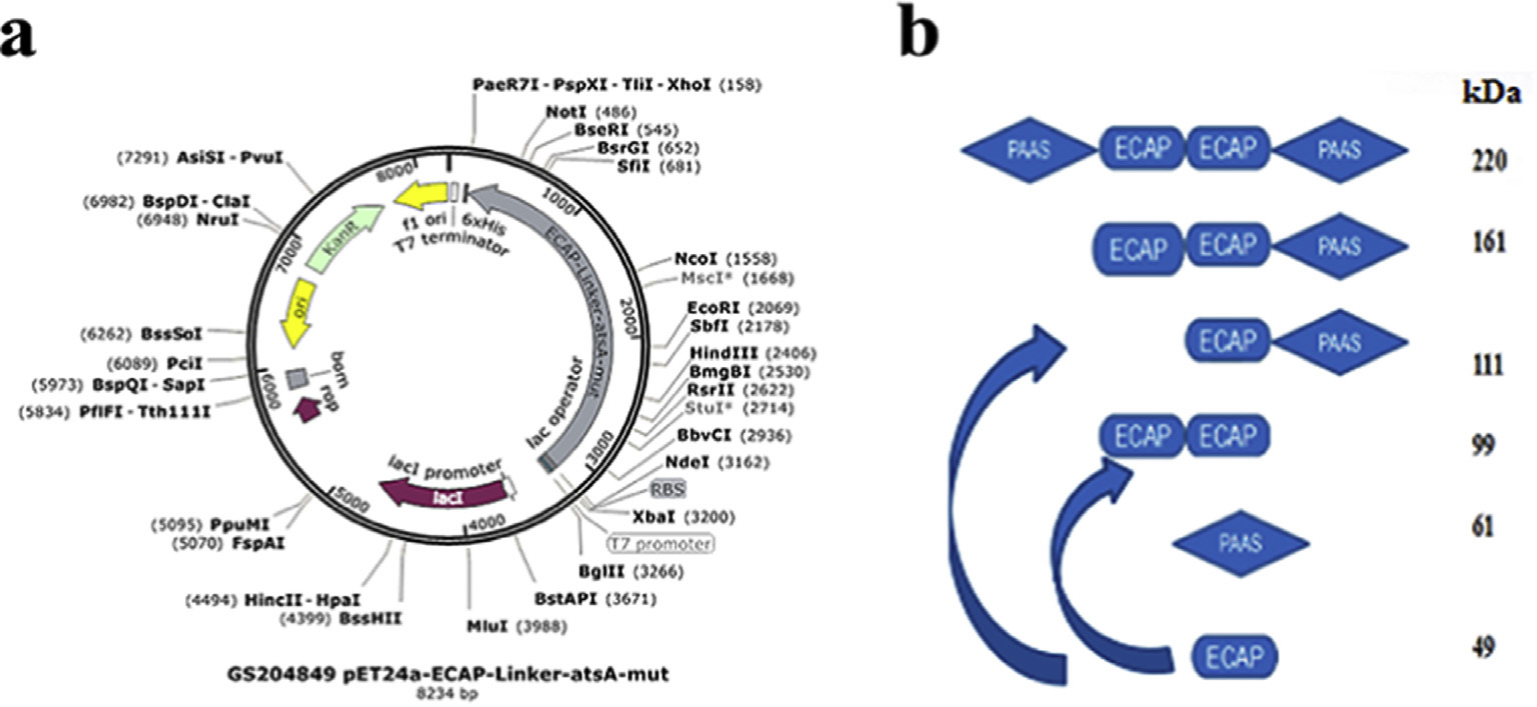
The expression vector map and active proteolytic fragments of fused forms. (a) The expression vector map. (b) Active proteolytic fragments of fused forms.

The data in this article provides information on how to develop an experimental system for HTP screening of mutants through the comparison of the activity ratios of an applicable enzyme and its mutants to a suitable enzyme tag in cell lysates of their fused forms (Fig. 1, Fig. 2, Fig. 3, Fig. 4 and Table 1, Table 2, Table 3, Table 4, Table 5, Table 6, Table 7). Data supported validity of the proposed strategy and the advantage to recognize the positive mutant in each pair of PAAS/mutants during HTP screening and elucidate the sequence-activity relationship of PAAS in HTP mode (Fig. 5) (see Scheme 1).

**Table 1:** Thresholds of ECAP activities in cell lysates. Data for 140 individual clones transformed with the blank plasmid.

PNS (405 nm)			4NNPP (450 nm)			Activity Ratio^a^
ΔA/min	After correction ΔA/min	Activity on 4NPS (U/L)	ΔA/min	After correction ΔA/min	Activity on 4NNPP (U/L)	
−0.00184	−0.00181	−1.57	0.00050	0.00085	0.47	−3.36
−0.00165	−0.00163	−1.42	0.00045	0.00077	0.42	−3.34
−0.00112	−0.00109	−0.95	0.00050	0.00071	0.39	−2.41
−0.00113	−0.00110	−0.96	0.00045	0.00067	0.37	−2.59
−0.00076	−0.00073	−0.64	0.00050	0.00064	0.36	−1.79
−0.00183	−0.00181	−1.57	0.00029	0.00064	0.35	−4.47
−0.00118	−0.00116	−1.01	0.00040	0.00062	0.34	−2.92
−0.00106	−0.00104	−0.90	0.00039	0.00059	0.33	−2.77
−0.00100	−0.00098	−0.85	0.00039	0.00058	0.32	−2.65
−0.00131	−0.00129	−1.12	0.00033	0.00058	0.32	−3.53
−0.00104	−0.00102	−0.88	0.00035	0.00054	0.30	−2.95
−0.00117	−0.00116	−1.00	0.00030	0.00052	0.29	−3.48
−0.00122	−0.00120	−1.04	0.00029	0.00052	0.29	−3.63
−0.00112	−0.00110	−0.96	0.00031	0.00052	0.29	−3.32
−0.00077	−0.00075	−0.65	0.00037	0.00052	0.29	−2.28
−0.00087	−0.00085	−0.74	0.00035	0.00051	0.28	−2.63
−0.00110	−0.00108	−0.94	0.00030	0.00051	0.28	−3.35
−0.00098	−0.00096	−0.84	0.00032	0.00050	0.28	−3.01
−0.00115	−0.00114	−0.99	0.00028	0.00050	0.28	−3.58
−0.00089	−0.00087	−0.76	0.00033	0.00050	0.27	−2.77
−0.00088	−0.00086	−0.75	0.00033	0.00049	0.27	−2.75
−0.00056	−0.00054	−0.47	0.00038	0.00049	0.27	−1.74
−0.00082	−0.00080	−0.69	0.00033	0.00048	0.27	−2.61
−0.00109	−0.00108	−0.94	0.00026	0.00047	0.26	−3.60
−0.00107	−0.00106	−0.92	0.00026	0.00047	0.26	−3.57
−0.00091	−0.00089	−0.78	0.00029	0.00046	0.26	−3.03
−0.00110	−0.00109	−0.94	0.00025	0.00046	0.26	−3.69
−0.00047	−0.00045	−0.39	0.00037	0.00046	0.26	−1.54
−0.00011	−0.00008	−0.07	0.00044	0.00046	0.25	−0.29
−0.00102	−0.00100	−0.87	0.00026	0.00046	0.25	−3.46
−0.00064	−0.00062	−0.54	0.00033	0.00045	0.25	−2.17
−0.00083	−0.00081	−0.70	0.00029	0.00045	0.25	−2.85
−0.00063	−0.00061	−0.53	0.00032	0.00044	0.24	−2.19
−0.00101	−0.00099	−0.86	0.00025	0.00044	0.24	−3.59
−0.00105	−0.00104	−0.90	0.00024	0.00044	0.24	−3.76
−0.00028	−0.00026	−0.23	0.00038	0.00044	0.24	−0.94
−0.00038	−0.00036	−0.31	0.00035	0.00043	0.24	−1.33
−0.00036	−0.00034	−0.30	0.00035	0.00042	0.23	−1.27
−0.00074	−0.00072	−0.63	0.00028	0.00042	0.23	−2.69
−0.00049	−0.00047	−0.41	0.00033	0.00042	0.23	−1.77
−0.00038	−0.00036	−0.31	0.00035	0.00042	0.23	−1.36
−0.00080	−0.00078	−0.68	0.00026	0.00042	0.23	−2.97
−0.00068	−0.00067	−0.58	0.00028	0.00041	0.23	−2.55
−0.00063	−0.00061	−0.53	0.00029	0.00041	0.23	−2.34
−0.00048	−0.00046	−0.40	0.00032	0.00041	0.23	−1.78
−0.00078	−0.00077	−0.67	0.00025	0.00039	0.22	−3.07
−0.00105	−0.00104	−0.91	0.00019	0.00039	0.22	−4.20
−0.00100	−0.00099	−0.86	0.00019	0.00038	0.21	−4.09
−0.00071	−0.00069	−0.60	0.00025	0.00038	0.21	−2.88
−0.00074	−0.00072	−0.63	0.00024	0.00038	0.21	−3.03
−0.00131	−0.00130	−1.13	0.00013	0.00038	0.21	−5.46
−0.00110	−0.00109	−0.95	0.00016	0.00037	0.21	−4.62
−0.00043	−0.00041	−0.36	0.00029	0.00037	0.21	−1.74
−0.00099	−0.00098	−0.85	0.00018	0.00037	0.20	−4.18
−0.00058	−0.00057	−0.49	0.00025	0.00036	0.20	−2.45
−0.00066	−0.00065	−0.57	0.00024	0.00036	0.20	−2.83
−0.00034	−0.00032	−0.28	0.00029	0.00035	0.20	−1.42
−0.00072	−0.00071	−0.61	0.00022	0.00035	0.20	−3.14
−0.00060	−0.00059	−0.51	0.00024	0.00035	0.19	−2.64
−0.00052	−0.00050	−0.44	0.00025	0.00034	0.19	−2.31
−0.00045	−0.00044	−0.38	0.00025	0.00034	0.19	−2.03
−0.00074	−0.00072	−0.63	0.00020	0.00034	0.19	−3.36
−0.00076	−0.00075	−0.65	0.00019	0.00034	0.19	−3.53
−0.00073	−0.00072	−0.62	0.00019	0.00033	0.18	−3.43
−0.00048	−0.00047	−0.41	0.00024	0.00033	0.18	−2.25
−0.00067	−0.00066	−0.58	0.00019	0.00032	0.18	−3.27
−0.00037	−0.00036	−0.31	0.00025	0.00032	0.17	−1.78
−0.00037	−0.00036	−0.31	0.00025	0.00032	0.17	−1.78
−0.00022	−0.00020	−0.18	0.00027	0.00031	0.17	−1.01
−0.00025	−0.00024	−0.21	0.00026	0.00031	0.17	−1.21
−0.00054	−0.00052	−0.46	0.00021	0.00031	0.17	−2.66
−0.00052	−0.00051	−0.44	0.00021	0.00031	0.17	−2.59
−0.00050	−0.00049	−0.42	0.00021	0.00030	0.17	−2.53
−0.00035	−0.00034	−0.30	0.00024	0.00030	0.17	−1.77
−0.00085	−0.00085	−0.74	0.00014	0.00030	0.16	−4.47
−0.00065	−0.00064	−0.56	0.00017	0.00030	0.16	−3.42
−0.00045	−0.00044	−0.38	0.00021	0.00030	0.16	−2.36
−0.00029	−0.00028	−0.24	0.00024	0.00029	0.16	−1.50
−0.00034	−0.00032	−0.28	0.00023	0.00029	0.16	−1.75
−0.00040	−0.00039	−0.34	0.00021	0.00028	0.16	−2.14
−0.00020	−0.00019	−0.16	0.00025	0.00028	0.16	−1.03
−0.00028	−0.00027	−0.23	0.00023	0.00028	0.16	−1.51
−0.00033	−0.00031	−0.27	0.00022	0.00028	0.15	−1.77
−0.00037	−0.00036	−0.31	0.00021	0.00028	0.15	−2.03
−0.00055	−0.00054	−0.47	0.00017	0.00028	0.15	−3.09
−0.00035	−0.00034	−0.30	0.00021	0.00028	0.15	−1.95
−0.00015	−0.00014	−0.12	0.00025	0.00027	0.15	−0.80
−0.00005	−0.00003	−0.03	0.00026	0.00027	0.15	−0.17
−0.00009	−0.00008	−0.07	0.00025	0.00027	0.15	−0.44
−0.00033	−0.00032	−0.27	0.00021	0.00027	0.15	−1.83
−0.00013	−0.00011	−0.10	0.00025	0.00027	0.15	−0.66
−0.00051	−0.00050	−0.43	0.00017	0.00027	0.15	−2.92
−0.00012	−0.00010	−0.09	0.00025	0.00027	0.15	−0.61
−0.00045	−0.00043	−0.38	0.00018	0.00027	0.15	−2.57
−0.00045	−0.00043	−0.38	0.00018	0.00027	0.15	−2.57
−0.00053	−0.00052	−0.45	0.00016	0.00026	0.15	−3.09
−0.00047	−0.00046	−0.40	0.00017	0.00026	0.14	−2.78
−0.00055	−0.00055	−0.47	0.00015	0.00026	0.14	−3.31
−0.00029	−0.00028	−0.24	0.00020	0.00026	0.14	−1.72
−0.00029	−0.00028	−0.24	0.00020	0.00026	0.14	−1.72
−0.00037	−0.00036	−0.31	0.00018	0.00025	0.14	−2.26
−0.00016	−0.00015	−0.13	0.00022	0.00025	0.14	−0.95
−0.00021	−0.00020	−0.17	0.00021	0.00025	0.14	−1.25
−0.00025	−0.00024	−0.21	0.00020	0.00025	0.14	−1.54
−0.00010	−0.00009	−0.08	0.00023	0.00025	0.14	−0.55
−0.00056	−0.00056	−0.48	0.00014	0.00024	0.13	−3.60
−0.00031	−0.00030	−0.26	0.00018	0.00024	0.13	−1.95
−0.00040	−0.00039	−0.34	0.00016	0.00024	0.13	−2.57
−0.00015	−0.00014	−0.12	0.00021	0.00024	0.13	−0.94
−0.00025	−0.00023	−0.20	0.00018	0.00023	0.13	−1.62
−0.00062	−0.00061	−0.53	0.00011	0.00023	0.12	−4.26
−0.00047	−0.00046	−0.40	0.00014	0.00023	0.12	−3.24
−0.00046	−0.00046	−0.40	0.00014	0.00022	0.12	−3.20
−0.00026	−0.00025	−0.22	0.00017	0.00022	0.12	−1.79
−0.00035	−0.00035	−0.30	0.00015	0.00022	0.12	−2.45
−0.00011	−0.00010	−0.09	0.00019	0.00021	0.12	−0.73
−0.00010	−0.00009	−0.08	0.00019	0.00021	0.12	−0.67
−0.00053	−0.00052	−0.45	0.00011	0.00021	0.12	−3.92
−0.00035	−0.00035	−0.30	0.00014	0.00020	0.11	−2.68
−0.00033	−0.00032	−0.28	0.00014	0.00020	0.11	−2.53
−0.00051	−0.00050	−0.44	0.00010	0.00020	0.11	−4.03
−0.00045	−0.00045	−0.39	0.00011	0.00020	0.11	−3.61
−0.00003	−0.00002	−0.01	0.00018	0.00019	0.10	−0.14
−0.00001	0.00000	0.00	0.00018	0.00018	0.10	0.01
−0.00015	−0.00014	−0.12	0.00015	0.00018	0.10	−1.18
−0.00045	−0.00044	−0.38	0.00009	0.00018	0.10	−3.95
−0.00018	−0.00017	−0.15	0.00014	0.00017	0.09	−1.60
0.00021	0.00022	0.19	0.00021	0.00017	0.09	2.06
−0.00022	−0.00021	−0.18	0.00013	0.00017	0.09	−1.97
−0.00014	−0.00013	−0.11	0.00013	0.00015	0.08	−1.33
−0.00005	−0.00005	−0.04	0.00014	0.00015	0.08	−0.50
−0.00004	−0.00003	−0.02	0.00014	0.00014	0.08	−0.31
−0.00047	−0.00047	−0.41	0.00005	0.00013	0.07	−5.48
−0.00031	−0.00030	−0.27	0.00007	0.00013	0.07	−3.66
0.00004	0.00004	0.04	0.00014	0.00013	0.07	0.54
0.00047	0.00048	0.42	0.00018	0.00009	0.05	8.24
0.00056	0.00057	0.50	0.00019	0.00008	0.05	10.74
−0.00015	−0.00014	−0.12	0.00005	0.00007	0.04	−3.08
0.00073	0.00074	0.64	0.00016	0.00003	0.01	44.67
0.00055	0.00056	0.48	0.00005	−0.00006	−0.03	−14.73

a Activity ratio was the activity of PAAS/mutant to that of ECAP in their fused form.

**Table 2:** Activity ratios of three fused forms *via* different linkers.

	Activity by SSA^b^			Activity by SDESA^c^		
	Activity on 4NPS (kU/g)	Activity on 4NNPP (kU/g)	Activity Ratio^a^	Activity on 4NPS (kU/g)	Activity on 4NNPP (kU/g)	Activity Ratio^a^
Cell lysate (*n* = 3)						
ECAP-dHis-PAAS	2.17 ± 0.532	0.36 ± 0.082	6.01 ± 0.597	2.04 ± 0.415	0.34 ± 0.072	6.07 ± 0.522
ECAP-LHis-PAAS	2.50 ± 0.622	0.40 ± 0.076	6.27 ± 0.710	2.55 ± 0.430	0.40 ± 0.091	6.39 ± 0.403
ECAP-LL-PAAS	2.28 ± 0.567	0.36 ± 0.077	6.25 ± 0.621	2.64 ± 0.397	0.43 ± 0.077	6.10 ± 0.389
Purified enzyme (*n* = 3)						
ECAP-dHis-PAAS	13.57 ± 3.02	2.22 ± 0.470	6.11 ± 0.457	12.67 ± 1.89	2.05 ± 0.402	6.17 ± 0.321
ECAP-LHis-PAAS	13.33 ± 2.16	2.14 ± 0.389	6.24 ± 0.397	12.00 ± 2.785	1.85 ± 0.5775	6.47 ± 0.774
ECAP-LL-PAAS	12.73 ± 2.03	2.03 ± 0.421	6.29 ± 0.687	12.54 ± 2.574	2.00 ± 0.510	6.26 0.440

a Activities were determined by SSA/SDESA, with proteins quantified by Bradford method, activity ratio was the activity of PAAS/mutant to that of ECAP in their fused form.

b SSA indicates separate single assay, one-by-one, in two solution.

c SDESA indicates spectrophotometric-dual-enzyme-simultaneous-assay.

**Table 3:** Comparison of two methods for cell lysis.

Mutants^a^	Alkline lysis (*n* = 6)		Sonication treatment (*n* = 6)	
	PNS/4NNPP	Relative value^b^	PNS/4NNPP	Relative value^b^
ECAP-PAAS	2.06 ± 0.03	42.18	1.96 ± 0.04	38.44
ECAP-M72L	1.68 ± 0.10	34.31	1.45 ± 0.11	28.33
ECAP-M72T	1.44 ± 0.05	29.36	1.81 ± 0.14	35.37
ECAP-M72Q	1.32 ± 0.07	26.95	2.00 ± 0.08	39.16
ECAP-M72W	1.19 ± 0.01	24.37	1.41 ± 0.04	27.54
ECAP-M72V	1.10 ± 0.08	22.57	0.86 ± 0.03	16.76
ECAP-M72I	1.02 ± 0.09	20.92	1.35 ± 0.14	26.51
ECAP-M72A	0.92 ± 0.03	18.81	1.23 ± 0.12	24.19
ECAP-M72S	0.69 ± 0.04	14.20	0.55 ± 0.03	10.76
ECAP-M72P	0.54 ± 0.02	11.07	0.55 ± 0.04	10.72
ECAP-M72C	0.49 ± 0.01	10.07	0.76 ± 0.02	14.87
ECAP-M72H	0.36 ± 0.04	7.39	0.43 ± 0.01	8.45
ECAP-M72N	0.28 ± 0.03	5.73	0.40 ± 0.02	7.91
ECAP-M72Y	0.26 ± 0.04	5.22	0.69 ± 0.04	13.49
ECAP-M72F	0.25 ± 0.04	5.17	0.22 ± 0.01	4.39
ECAP-M72D	0.25 ± 0.03	5.02	0.32 ± 0.01	6.31
ECAP-M72E	0.15 ± 0.02	3.10	0.17 ± 0.01	3.39
ECAP-M72K	0.09 ± 0.03	1.87	0.12 ± 0.00	2.34
ECAP-M72G	0.08 ± 0.03	1.65	0.10 ± 0.01	1.86
ECAP-M72R	0.05 ± 0.02	1.00	0.05 ± 0.00	1.00

a Paired *t*-test was applied to the comparison of relative values of activity ratios to ECAP between two lysis methods, giving *t* = 1.496, *P* = 0.15.

b Relative value was derived as the activity ratio to that of M72R.

**Table 4:** Association of activity ratios in cell lysates of the fused forms with specific activities of their non-fused counterparts based on ITA.

Mutants	Specific activity by ITA (kU/g)	Relative value^a^	Log_10_(Specific activity by ITA)	log_10_ (Relative value)
ECAP-PAAS	14.60	54.36	1.16	1.74
ECAP-M72L	8.35	35.37	0.92	1.55
ECAP-M72T	8.18	39.16	0.91	1.59
ECAP-M72Q	7.10	28.33	0.85	1.45
ECAP-M72W	7.07	26.52	0.85	1.42
ECAP-M72V	5.06	27.55	0.70	1.44
ECAP-M72A	4.92	24.19	0.69	1.38
ECAP-M72I	4.96	16.76	0.70	1.22
ECAP-M72S	3.68	14.87	0.57	1.17
ECAP-M72C	3.46	10.76	0.54	1.03
ECAP-M72P	3.60	13.49	0.56	1.13
ECAP-M72H	2.41	10.72	0.38	1.03
ECAP-M72Y	1.05	8.45	0.02	0.93
ECAP-M72D	1.84	7.91	0.27	0.90
ECAP-M72E	0.45	6.31	−0.34	0.80
ECAP-M72N	1.42	4.39	0.15	0.64
ECAP-M72G	1.18	3.40	0.07	0.53
ECAP-M72F	0.31	2.34	−0.50	0.37
ECAP-M72K	0.59	1.86	−0.23	0.27
ECAP-M72R	0.12	1.00	−0.93	0.00

a Relative value was derived as the activity ratio to that of M72R.

**Table 5:** Association of activity ratios in cell lysates of the fused forms with specific activities of their non-fused counterparts after affinity purification.

	Activity of non-fused PAAS/mutant		Activity by SSA of ECAP-PAAS/mutant^c^		Activity by SDESA of ECAP-PAAS/mutant^d^	
	Specific activity (kU/g)	Relative value^b^	Activity Ratio^a^	Relative value^b^	Activity Ratio^a^	Relative value^b^
PAAS	14.09	12.3	6.24	11.3	5.20	12.8
M72Q	4.70	4.1	2.05	3.7	1.60	3.9
G138S	3.16	2.8	1.12	2.0	0.78	1.9
M72D	1.15	1.0	0.55		0.405	1.0

a Activities were determined by SSA/SDESA, with proteins quantified by the Bradford method, activity ratio was the activity of PAAS/mutant to that of ECAP in their fused form.

b Relative value was derived as the ratio to that ofM72D in the non-fused or fused form.

c SSA indicates separate single assay, one-by-one, in two solution.

d SDESA indicates spectrophotometric-dual-enzyme-simultaneous-assay.

**Table 6:** Activities and activity ratios in cell lysates of tested PAAS/mutants in fused forms by either SSA or SDESA.

	Activity by SSA of ECAP -PAAS/mutants^c^ (*n* = 120)				Activity by SDESA of ECAP -PAAS/mutants^d^ (*n* = 120)			
	Activity on 4NPS (kU/g)	Activity on 4NNPP (kU/g)	Activity Ratio^a^	Relative value^b^	Activity on 4NPS (kU/g)	Activity on 4NNPP (kU/g)	Activity Ratio^a^	Relative value^b^
ECAP-PAAS								
Mean	0.024	0.004	6.237	11.3	0.020	0.004	5.201	12.8
SD	0.006	0.001	1.229		0.004	0.001	1.041	
cv	23%	19%	20%		21%	18%	20%	
ECAP-M72Q								
Mean	0.014	0.007	2.052	3.7	0.010	0.0065	1.600	3.9
SD	0.005	0.002	0.322		0.004	0.002	0.232	
cv	35%	27%	16%		35%	29%	15%	
ECAP-G138S								
Mean	0.009	0.008	1.117	2.0	0.007	0.008	0.780	1.9
SD	0.003	0.003	0.228		0.002	0.003	0.129	
cv	33%	41%	20%		34%	31%	17%	
ECAP-M72D								
mean	0.006	0.011	0.553	1.0	0.004	0.010	0.405	1.0
SD	0.001	0.003	0.079		0.001	0.003	0.053	
cv	19%	27%	14%		21%	28%	13%	

a Activities were determined by SSA/SDESA, with proteins quantified by Bradford method, activity ratio was the activity of PAAS/mutant to that of ECAP in their fused form.

b Relative value was derived as the ratio to that of M72D in fused form.

c SSA indicates separate single assay, one-by-one, in two solution.

d SDESA indicates spectrophotometric-dual-enzyme-simultaneous-assay.

**Table 7:** One-way ANOVA for statistical analysis of activity ratios determined in HTP mode for paired fused PAAS/mutants.

	ECAP-G138S						ECAP-M72Q					
	ANOVA Result of 4PNS/4NNPP between SSA and SDESA			ANOVA Result of 4PNS/4NNPP ratio to M72D between SSA and SDESA			ANOVA Result of 4PNS/4NNPP between SSA and SDESA			ANOVA Result of 4PNS/4NNPP ratio to M72D between SSA and SDESA		
	Between Groups	Within Groups	Total	Between Groups	Within Groups	Total	Between Groups	Within Groups	Total	Between Groups	Within Groups	Total
Sum of Squares	6.316	7.536	13.852	0.529	29.854	30.383	12.23	18.749	30.979	3.33	79.426	82.756
df	1	220	221	1	220	221	1	238	239	1	238	239
Mean Squares	6.316	0.034		0.529	0.136		12.23	0.079		3.33	0.334	
F	184.37			3.895			155.251			9.978		
Sig.	0.000			0.050			0.000			0.002		
	ECAP-PAAS						ECAP-M72D					
	ANOVA Result of 4PNS/4NNPP between SSA and SDESA			ANOVA Result of 4PNS/4NNPP ratio to M72D between SSA and SDESA			ANOVA Result of 4PNS/4NNPP between SSA and SDESA			ANOVA Result of 4PNS/4NNPP ratio to M72D between SSA and SDESA		
	Between Groups	Within Groups	Total	Between Groups	Within Groups	Total	Between Groups	Within Groups	Total	Between Groups	Within Groups	Total
Sum of Squares	60.137	287.826	347.963	133.731	1280.626	1414.357	1.206	0.99	2.195	0	4.114	4.114
df	1	222	223	1	222	223	1	220	221	1	220	221
Mean Squares	60.137	1.297		133.731	5.769		1.206	0.004		0	0.019	
F	46.384			23.183			268.029			0		
Sig.	0.000			0.000			0.000			0.993		

SSA: separate single assay. SDESA: spectrophotometric-dual-enzyme-simultaneous-assay.

The lane of the same label in figures stood for the same sample, as follows. M: molecular weight markers; 0. Transformed with empty plasmid; 1. Purified ECAP; 2. Purified PAAS; 3. Purified ECAP-dHis-PAAS; 4. Purified ECAP-LHis-PAAS; 5. Purified ECAP-LL-PAAS; 6. ECAP-dHis-PAAS in lysate; 7. ECAP-LHis-PAAS in lysate; 8. ECAP-LL-PAAS in lysate.

## 2 Experimental design, materials and methods

### 2.1 Experimental design

The comparison of the activity ratios of an applicable enzyme and its mutants to a suitable enzyme tag in cell lysates of their fused forms for HTP screening of mutants requires both the negligible or consistent impacts of the enzyme tag on the activities of enzyme/mutants and the proportionality of the activities of the enzyme tag in cell lysates of the fused enzyme/mutants to protein quantities of the active forms of the fused enzyme/mutants in both their native and partially-fragmented fused states. With ECAP as the tag fused to the *N*-terminus of PAAS and its mutants *via* a flexible linker, the proposed strategy was tested.

## 3 Materials and methods

For each fused enzyme/mutant, an individual clone was transferred into 0.50 mL LB medium in 48-well microplate for amplification in 12 h at 37 °C and 180 rpm till optical density of 0.4–0.6 at 600 nm. Afterwards, each enzyme/mutant was induced for expression with 1.0 mM IPTG for 21 h at 15 °C. The lysates of fused mutants were prepared through alkaline lysis, unless otherwise stated. In detail, cell suspension of 20 μL from a well was transferred to a new well for mixing with 180 μL of the alkaline lysis buffer (1.0 M Tris-HCl at pH 9.0, plus 1.0 mM PMSF and 2.5 mM 4-aminobenzamidine) in 96-well microplates; the resulting mixture was agitated rapidly on Qilinbeier QB-9001 agitator for 4 h at room temperature to yield a cell lysate.

The substrate solution containing 2.0 mM 4NPS and/or 0.20 mM 4NNPP was utilized to monitor the absorbance change at 405 and/or 450 nm (The substrate solution containing both substrates was utilized for spectrophotometric-dual-enzyme-simultaneous-assay (SDESA) of ECAP and PAAS/mutant [2,3]). For HTP assay of enzyme activity(ies), cell lysate of 20 μL was mixed with a substrate solution of 180 μL in 96-well microplates. The mixtures in wells were agitated for 2.0 min at room tempearture; after a total lagging time of 4.0 min, the absorbance of each well was recorded in 30 min at room tempearture to estimate initial rate for enzyme activity by linear regression with the preset absorptivity of 12 (mmol)^−1^·L^−1^·cm^−1^ for 4-nitrophenol and 19 (mmol)^−1^·L^−1^·cm^−1^ for 4-nitro-1-naphthol considering the light path. The change of absorbance of no less than 0.003 in 10 min was taken as the detection limit of enzyme activity, after the correction of the overlapped absorbance of chromogenic products during SDESA.

The activity ratio of PAAS/mutant to ECAP was the percentage of PAAS/mutant activity on 4NPS to ECAP activity on 4NNPP. Receiver-operating-characteristic (ROC) analysis yielded the area-under-the-curve (AUC) for the recognition of the one of higher activity in a pair.
