# Supplementary material for: Data for high-throughput screening of enzyme mutants by comparison of their activity ratios to an enzyme tag
Source: Data Brief. 2019 Dec 13;28:104985. doi: 10.1016/j.dib.2019.104985 (PMC6931123; doi:10.1016/j.dib.2019.104985)
Supplement: Multimedia component 1 [file mmc1.doc]

**Table 1-2: Data for 598 individual clones of the fused mutants after saturation mutagenesis of PAAS at M72.**

| PNS (405 nm) | | | 4NNPP (450 nm) | | | Activity Ratio ^a^ |
| --- | --- | --- | --- | --- | --- | --- |
| ΔA/min | After correction ΔA/min | Activity on 4NPS (U/L) | ΔA/min | After correction ΔA/min | Activity on  4NNPP (U/L) |
| -0.00074 | 0.00230 | 2.00 | 0.05189 | 0.05203 | 28.75 | 0.07 |
| 0.00404 | 0.00696 | 6.05 | 0.04976 | 0.04900 | 27.07 | 0.22 |
| -0.00076 | 0.00201 | 1.75 | 0.04728 | 0.04742 | 26.20 | 0.07 |
| 0.01325 | 0.01605 | 13.96 | 0.04783 | 0.04532 | 25.04 | 0.56 |
| 0.00040 | 0.00304 | 2.65 | 0.04504 | 0.04496 | 24.84 | 0.11 |
| -0.00027 | 0.00218 | 1.90 | 0.04181 | 0.04186 | 23.13 | 0.08 |
| 0.00075 | 0.00320 | 2.78 | 0.04170 | 0.04156 | 22.96 | 0.12 |
| 0.02748 | 0.03021 | 26.27 | 0.04652 | 0.04131 | 22.82 | 1.15 |
| 0.00429 | 0.00670 | 5.83 | 0.04125 | 0.04043 | 22.34 | 0.26 |
| 0.00042 | 0.00278 | 2.41 | 0.04019 | 0.04011 | 22.16 | 0.11 |
| 0.00091 | 0.00322 | 2.80 | 0.03942 | 0.03925 | 21.68 | 0.13 |
| 0.00030 | 0.00260 | 2.26 | 0.03929 | 0.03924 | 21.68 | 0.10 |
| 0.00237 | 0.00466 | 4.05 | 0.03918 | 0.03873 | 21.40 | 0.19 |
| 0.00394 | 0.00624 | 5.43 | 0.03933 | 0.03859 | 21.32 | 0.25 |
| 0.00130 | 0.00356 | 3.09 | 0.03852 | 0.03828 | 21.15 | 0.15 |
| 0.00341 | 0.00568 | 4.94 | 0.03875 | 0.03810 | 21.05 | 0.23 |
| 0.00325 | 0.00551 | 4.79 | 0.03867 | 0.03805 | 21.02 | 0.23 |
| -0.00169 | 0.00052 | 0.45 | 0.03767 | 0.03799 | 20.99 | 0.02 |
| -0.00095 | 0.00124 | 1.08 | 0.03748 | 0.03766 | 20.81 | 0.05 |
| -0.00133 | 0.00087 | 0.75 | 0.03740 | 0.03766 | 20.80 | 0.04 |
| 0.00142 | 0.00363 | 3.16 | 0.03778 | 0.03751 | 20.72 | 0.15 |
| 0.02163 | 0.02405 | 20.92 | 0.04143 | 0.03733 | 20.63 | 1.01 |
| -0.00125 | 0.00093 | 0.81 | 0.03706 | 0.03730 | 20.61 | 0.04 |
| 0.02529 | 0.02775 | 24.13 | 0.04198 | 0.03720 | 20.55 | 1.17 |
| -0.00052 | 0.00165 | 1.43 | 0.03707 | 0.03717 | 20.54 | 0.07 |
| -0.00065 | 0.00151 | 1.31 | 0.03690 | 0.03702 | 20.45 | 0.06 |
| -0.00116 | 0.00098 | 0.85 | 0.03645 | 0.03667 | 20.26 | 0.04 |
| 0.00153 | 0.00369 | 3.21 | 0.03679 | 0.03650 | 20.17 | 0.16 |
| 0.00231 | 0.00447 | 3.89 | 0.03686 | 0.03642 | 20.12 | 0.19 |
| -0.00010 | 0.00204 | 1.77 | 0.03640 | 0.03641 | 20.12 | 0.09 |
| -0.00095 | 0.00117 | 1.02 | 0.03623 | 0.03641 | 20.12 | 0.05 |
| -0.00037 | 0.00176 | 1.53 | 0.03631 | 0.03638 | 20.10 | 0.08 |
| 0.00843 | 0.01065 | 9.26 | 0.03794 | 0.03634 | 20.08 | 0.46 |
| -0.00269 | -0.00059 | -0.51 | 0.03581 | 0.03632 | 20.07 | -0.03 |
| 0.00084 | 0.00298 | 2.59 | 0.03647 | 0.03631 | 20.06 | 0.13 |
| -0.00019 | 0.00193 | 1.68 | 0.03625 | 0.03629 | 20.05 | 0.08 |
| -0.00137 | 0.00074 | 0.64 | 0.03594 | 0.03620 | 20.00 | 0.03 |
| -0.00116 | 0.00095 | 0.82 | 0.03594 | 0.03616 | 19.98 | 0.04 |
| -0.00046 | 0.00165 | 1.44 | 0.03603 | 0.03612 | 19.96 | 0.07 |
| -0.00110 | 0.00100 | 0.87 | 0.03584 | 0.03605 | 19.92 | 0.04 |
| 0.00094 | 0.00307 | 2.67 | 0.03622 | 0.03604 | 19.91 | 0.13 |
| 0.00237 | 0.00450 | 3.91 | 0.03645 | 0.03600 | 19.89 | 0.20 |
| 0.00074 | 0.00286 | 2.48 | 0.03613 | 0.03600 | 19.89 | 0.12 |
| 0.00203 | 0.00416 | 3.62 | 0.03633 | 0.03595 | 19.86 | 0.18 |
| 0.00061 | 0.00272 | 2.37 | 0.03602 | 0.03590 | 19.83 | 0.12 |
| -0.00108 | 0.00101 | 0.88 | 0.03566 | 0.03586 | 19.81 | 0.04 |
| 0.00040 | 0.00250 | 2.18 | 0.03594 | 0.03586 | 19.81 | 0.11 |
| 0.00004 | 0.00214 | 1.86 | 0.03579 | 0.03578 | 19.77 | 0.09 |
| 0.00012 | 0.00222 | 1.93 | 0.03577 | 0.03575 | 19.75 | 0.10 |
| -0.00094 | 0.00113 | 0.98 | 0.03537 | 0.03555 | 19.64 | 0.05 |
| -0.00074 | 0.00134 | 1.16 | 0.03539 | 0.03553 | 19.63 | 0.06 |
| 0.00075 | 0.00282 | 2.46 | 0.03548 | 0.03533 | 19.52 | 0.13 |
| -0.00099 | 0.00106 | 0.93 | 0.03509 | 0.03528 | 19.49 | 0.05 |
| -0.00061 | 0.00145 | 1.26 | 0.03514 | 0.03526 | 19.48 | 0.06 |
| -0.00148 | 0.00056 | 0.49 | 0.03492 | 0.03520 | 19.45 | 0.03 |
| -0.00129 | 0.00075 | 0.66 | 0.03495 | 0.03520 | 19.45 | 0.03 |
| 0.00163 | 0.00371 | 3.23 | 0.03541 | 0.03510 | 19.39 | 0.17 |
| 0.00220 | 0.00428 | 3.72 | 0.03548 | 0.03507 | 19.37 | 0.19 |
| -0.00155 | 0.00049 | 0.43 | 0.03475 | 0.03505 | 19.36 | 0.02 |
| -0.00002 | 0.00204 | 1.77 | 0.03500 | 0.03500 | 19.34 | 0.09 |
| -0.00120 | 0.00084 | 0.73 | 0.03475 | 0.03498 | 19.33 | 0.04 |
| 0.00042 | 0.00247 | 2.15 | 0.03505 | 0.03497 | 19.32 | 0.11 |
| 0.00063 | 0.00268 | 2.33 | 0.03508 | 0.03496 | 19.32 | 0.12 |
| -0.00087 | 0.00116 | 1.01 | 0.03475 | 0.03491 | 19.29 | 0.05 |
| -0.00084 | 0.00119 | 1.03 | 0.03459 | 0.03475 | 19.20 | 0.05 |
| 0.00062 | 0.00266 | 2.31 | 0.03483 | 0.03472 | 19.18 | 0.12 |
| 0.00128 | 0.00333 | 2.89 | 0.03495 | 0.03471 | 19.18 | 0.15 |
| 0.00174 | 0.00379 | 3.29 | 0.03500 | 0.03467 | 19.16 | 0.17 |
| 0.00337 | 0.00544 | 4.73 | 0.03529 | 0.03465 | 19.14 | 0.25 |
| 0.00713 | 0.00924 | 8.04 | 0.03595 | 0.03460 | 19.12 | 0.42 |
| -0.00127 | 0.00074 | 0.64 | 0.03433 | 0.03457 | 19.10 | 0.03 |
| 0.00163 | 0.00368 | 3.20 | 0.03483 | 0.03452 | 19.07 | 0.17 |
| 0.00240 | 0.00445 | 3.87 | 0.03494 | 0.03448 | 19.05 | 0.20 |
| 0.00006 | 0.00208 | 1.81 | 0.03448 | 0.03447 | 19.05 | 0.09 |
| -0.00010 | 0.00192 | 1.67 | 0.03435 | 0.03437 | 18.99 | 0.09 |
| -0.00010 | 0.00192 | 1.67 | 0.03431 | 0.03433 | 18.97 | 0.09 |
| 0.00195 | 0.00398 | 3.46 | 0.03465 | 0.03428 | 18.94 | 0.18 |
| -0.00075 | 0.00125 | 1.09 | 0.03410 | 0.03424 | 18.92 | 0.06 |
| 0.01986 | 0.02208 | 19.20 | 0.03800 | 0.03424 | 18.92 | 1.02 |
| 0.00339 | 0.00543 | 4.72 | 0.03487 | 0.03423 | 18.91 | 0.25 |
| 0.02129 | 0.02353 | 20.46 | 0.03821 | 0.03418 | 18.88 | 1.08 |
| -0.00081 | 0.00118 | 1.03 | 0.03401 | 0.03416 | 18.87 | 0.05 |
| 0.02023 | 0.02246 | 19.53 | 0.03798 | 0.03415 | 18.87 | 1.03 |
| -0.00010 | 0.00190 | 1.65 | 0.03410 | 0.03411 | 18.85 | 0.09 |
| -0.00051 | 0.00148 | 1.29 | 0.03398 | 0.03407 | 18.82 | 0.07 |
| 0.00210 | 0.00411 | 3.58 | 0.03442 | 0.03402 | 18.80 | 0.19 |
| -0.00068 | 0.00130 | 1.13 | 0.03388 | 0.03401 | 18.79 | 0.06 |
| 0.00225 | 0.00426 | 3.71 | 0.03440 | 0.03398 | 18.77 | 0.20 |
| 0.00156 | 0.00356 | 3.10 | 0.03425 | 0.03396 | 18.76 | 0.17 |
| -0.00079 | 0.00118 | 1.03 | 0.03375 | 0.03390 | 18.73 | 0.05 |
| -0.00015 | 0.00183 | 1.59 | 0.03383 | 0.03386 | 18.71 | 0.09 |
| 0.00191 | 0.00392 | 3.41 | 0.03422 | 0.03386 | 18.71 | 0.18 |
| -0.00071 | 0.00127 | 1.10 | 0.03370 | 0.03383 | 18.69 | 0.06 |
| 0.00122 | 0.00321 | 2.79 | 0.03393 | 0.03370 | 18.62 | 0.15 |
| -0.00102 | 0.00094 | 0.82 | 0.03349 | 0.03369 | 18.61 | 0.04 |
| -0.00072 | 0.00124 | 1.08 | 0.03352 | 0.03366 | 18.60 | 0.06 |
| 0.00067 | 0.00265 | 2.31 | 0.03374 | 0.03361 | 18.57 | 0.12 |
| 0.00240 | 0.00439 | 3.82 | 0.03406 | 0.03361 | 18.57 | 0.21 |
| 0.00031 | 0.00228 | 1.98 | 0.03363 | 0.03358 | 18.55 | 0.11 |
| -0.00062 | 0.00134 | 1.17 | 0.03344 | 0.03355 | 18.54 | 0.06 |
| 0.03394 | 0.03629 | 31.55 | 0.03998 | 0.03355 | 18.54 | 1.70 |
| 0.00032 | 0.00229 | 1.99 | 0.03360 | 0.03354 | 18.53 | 0.11 |
| 0.00049 | 0.00246 | 2.14 | 0.03360 | 0.03351 | 18.51 | 0.12 |
| -0.00098 | 0.00097 | 0.85 | 0.03329 | 0.03348 | 18.50 | 0.05 |
| 0.00179 | 0.00376 | 3.27 | 0.03369 | 0.03335 | 18.43 | 0.18 |
| -0.00079 | 0.00116 | 1.01 | 0.03319 | 0.03334 | 18.42 | 0.05 |
| 0.00004 | 0.00199 | 1.73 | 0.03330 | 0.03329 | 18.39 | 0.09 |
| -0.00114 | 0.00079 | 0.69 | 0.03306 | 0.03328 | 18.39 | 0.04 |
| -0.00022 | 0.00172 | 1.50 | 0.03321 | 0.03325 | 18.37 | 0.08 |
| -0.00059 | 0.00135 | 1.18 | 0.03313 | 0.03324 | 18.36 | 0.06 |
| 0.00191 | 0.00388 | 3.37 | 0.03358 | 0.03322 | 18.35 | 0.18 |
| -0.00181 | 0.00012 | 0.10 | 0.03285 | 0.03319 | 18.34 | 0.01 |
| 0.00200 | 0.00397 | 3.45 | 0.03356 | 0.03318 | 18.33 | 0.19 |
| 0.00102 | 0.00298 | 2.59 | 0.03336 | 0.03316 | 18.32 | 0.14 |
| 0.00163 | 0.00360 | 3.13 | 0.03347 | 0.03316 | 18.32 | 0.17 |
| -0.00138 | 0.00054 | 0.47 | 0.03285 | 0.03311 | 18.29 | 0.03 |
| -0.00068 | 0.00125 | 1.09 | 0.03297 | 0.03310 | 18.29 | 0.06 |
| -0.00090 | 0.00102 | 0.89 | 0.03292 | 0.03309 | 18.28 | 0.05 |
| -0.00110 | 0.00082 | 0.72 | 0.03288 | 0.03309 | 18.28 | 0.04 |
| -0.00037 | 0.00156 | 1.36 | 0.03292 | 0.03299 | 18.23 | 0.07 |
| -0.00056 | 0.00137 | 1.19 | 0.03287 | 0.03298 | 18.22 | 0.07 |
| -0.00028 | 0.00165 | 1.43 | 0.03287 | 0.03292 | 18.19 | 0.08 |
| -0.00061 | 0.00131 | 1.14 | 0.03277 | 0.03289 | 18.17 | 0.06 |
| 0.00125 | 0.00319 | 2.77 | 0.03310 | 0.03286 | 18.15 | 0.15 |
| 0.00026 | 0.00219 | 1.90 | 0.03290 | 0.03285 | 18.15 | 0.10 |
| 0.00256 | 0.00452 | 3.93 | 0.03333 | 0.03284 | 18.14 | 0.22 |
| 0.00028 | 0.00221 | 1.92 | 0.03289 | 0.03284 | 18.14 | 0.11 |
| -0.00122 | 0.00069 | 0.60 | 0.03256 | 0.03279 | 18.12 | 0.03 |
| 0.00181 | 0.00375 | 3.26 | 0.03311 | 0.03277 | 18.10 | 0.18 |
| 0.00065 | 0.00258 | 2.24 | 0.03289 | 0.03277 | 18.10 | 0.12 |
| -0.00028 | 0.00164 | 1.42 | 0.03268 | 0.03274 | 18.09 | 0.08 |
| -0.00029 | 0.00163 | 1.42 | 0.03266 | 0.03271 | 18.07 | 0.08 |
| 0.00230 | 0.00424 | 3.69 | 0.03314 | 0.03271 | 18.07 | 0.20 |
| -0.00062 | 0.00129 | 1.12 | 0.03258 | 0.03270 | 18.06 | 0.06 |
| 0.00237 | 0.00432 | 3.75 | 0.03314 | 0.03269 | 18.06 | 0.21 |
| 0.00255 | 0.00449 | 3.90 | 0.03311 | 0.03263 | 18.03 | 0.22 |
| -0.00042 | 0.00148 | 1.29 | 0.03251 | 0.03259 | 18.00 | 0.07 |
| 0.00076 | 0.00268 | 2.33 | 0.03273 | 0.03259 | 18.00 | 0.13 |
| 0.00031 | 0.00222 | 1.93 | 0.03260 | 0.03254 | 17.98 | 0.11 |
| -0.00099 | 0.00090 | 0.78 | 0.03221 | 0.03240 | 17.90 | 0.04 |
| -0.00181 | 0.00007 | 0.06 | 0.03202 | 0.03237 | 17.88 | 0.00 |
| 0.00225 | 0.00417 | 3.62 | 0.03277 | 0.03234 | 17.87 | 0.20 |
| -0.00100 | 0.00088 | 0.77 | 0.03215 | 0.03234 | 17.87 | 0.04 |
| -0.00028 | 0.00161 | 1.40 | 0.03224 | 0.03229 | 17.84 | 0.08 |
| 0.00099 | 0.00290 | 2.52 | 0.03248 | 0.03229 | 17.84 | 0.14 |
| -0.00045 | 0.00143 | 1.25 | 0.03219 | 0.03228 | 17.83 | 0.07 |
| 0.00116 | 0.00306 | 2.66 | 0.03248 | 0.03226 | 17.82 | 0.15 |
| 0.01090 | 0.01291 | 11.23 | 0.03427 | 0.03221 | 17.79 | 0.63 |
| -0.00143 | 0.00044 | 0.38 | 0.03190 | 0.03218 | 17.78 | 0.02 |
| -0.00040 | 0.00147 | 1.28 | 0.03207 | 0.03215 | 17.76 | 0.07 |
| 0.00102 | 0.00291 | 2.53 | 0.03233 | 0.03213 | 17.75 | 0.14 |
| -0.00049 | 0.00139 | 1.20 | 0.03204 | 0.03213 | 17.75 | 0.07 |
| 0.00222 | 0.00413 | 3.59 | 0.03252 | 0.03210 | 17.74 | 0.20 |
| 0.00149 | 0.00339 | 2.95 | 0.03234 | 0.03206 | 17.71 | 0.17 |
| -0.00119 | 0.00067 | 0.58 | 0.03179 | 0.03201 | 17.69 | 0.03 |
| -0.00065 | 0.00121 | 1.06 | 0.03183 | 0.03195 | 17.65 | 0.06 |
| 0.00037 | 0.00224 | 1.95 | 0.03194 | 0.03188 | 17.61 | 0.11 |
| 0.00165 | 0.00354 | 3.07 | 0.03217 | 0.03186 | 17.60 | 0.17 |
| -0.00011 | 0.00175 | 1.53 | 0.03183 | 0.03185 | 17.60 | 0.09 |
| 0.01493 | 0.01696 | 14.75 | 0.03467 | 0.03184 | 17.59 | 0.84 |
| 0.00022 | 0.00209 | 1.82 | 0.03187 | 0.03183 | 17.59 | 0.10 |
| 0.01990 | 0.02199 | 19.12 | 0.03551 | 0.03174 | 17.54 | 1.09 |
| 0.00037 | 0.00223 | 1.94 | 0.03180 | 0.03173 | 17.53 | 0.11 |
| -0.00037 | 0.00149 | 1.29 | 0.03163 | 0.03170 | 17.52 | 0.07 |
| -0.00070 | 0.00115 | 1.00 | 0.03151 | 0.03164 | 17.48 | 0.06 |
| -0.00075 | 0.00108 | 0.94 | 0.03137 | 0.03152 | 17.41 | 0.05 |
| 0.00013 | 0.00198 | 1.72 | 0.03154 | 0.03152 | 17.41 | 0.10 |
| 0.00169 | 0.00356 | 3.09 | 0.03183 | 0.03151 | 17.41 | 0.18 |
| 0.00171 | 0.00357 | 3.10 | 0.03175 | 0.03142 | 17.36 | 0.18 |
| -0.00087 | 0.00096 | 0.84 | 0.03122 | 0.03139 | 17.34 | 0.05 |
| 0.00067 | 0.00252 | 2.19 | 0.03148 | 0.03135 | 17.32 | 0.13 |
| 0.00122 | 0.00307 | 2.67 | 0.03153 | 0.03130 | 17.29 | 0.15 |
| 0.00156 | 0.00341 | 2.96 | 0.03158 | 0.03128 | 17.28 | 0.17 |
| -0.00108 | 0.00074 | 0.64 | 0.03099 | 0.03120 | 17.24 | 0.04 |
| 0.00101 | 0.00285 | 2.47 | 0.03137 | 0.03117 | 17.22 | 0.14 |
| 0.00175 | 0.00359 | 3.12 | 0.03148 | 0.03115 | 17.21 | 0.18 |
| 0.00100 | 0.00284 | 2.47 | 0.03133 | 0.03114 | 17.20 | 0.14 |
| 0.00094 | 0.00278 | 2.42 | 0.03130 | 0.03112 | 17.19 | 0.14 |
| -0.00086 | 0.00096 | 0.83 | 0.03094 | 0.03111 | 17.19 | 0.05 |
| 0.00094 | 0.00278 | 2.42 | 0.03128 | 0.03110 | 17.18 | 0.14 |
| 0.00003 | 0.00185 | 1.61 | 0.03110 | 0.03110 | 17.18 | 0.09 |
| -0.00076 | 0.00105 | 0.91 | 0.03095 | 0.03110 | 17.18 | 0.05 |
| -0.00010 | 0.00172 | 1.49 | 0.03107 | 0.03109 | 17.18 | 0.09 |
| -0.00064 | 0.00117 | 1.02 | 0.03097 | 0.03109 | 17.18 | 0.06 |
| 0.00089 | 0.00272 | 2.36 | 0.03124 | 0.03107 | 17.17 | 0.14 |
| 0.00169 | 0.00353 | 3.07 | 0.03137 | 0.03105 | 17.16 | 0.18 |
| 0.00187 | 0.00371 | 3.22 | 0.03140 | 0.03105 | 17.16 | 0.19 |
| 0.00084 | 0.00267 | 2.32 | 0.03121 | 0.03105 | 17.15 | 0.14 |
| 0.00109 | 0.00292 | 2.54 | 0.03122 | 0.03102 | 17.14 | 0.15 |
| -0.00087 | 0.00094 | 0.82 | 0.03076 | 0.03093 | 17.09 | 0.05 |
| 0.00187 | 0.00370 | 3.22 | 0.03128 | 0.03092 | 17.09 | 0.19 |
| -0.00021 | 0.00159 | 1.39 | 0.03084 | 0.03088 | 17.06 | 0.08 |
| 0.00073 | 0.00255 | 2.21 | 0.03101 | 0.03087 | 17.06 | 0.13 |
| 0.00030 | 0.00211 | 1.84 | 0.03092 | 0.03086 | 17.05 | 0.11 |
| 0.00028 | 0.00209 | 1.82 | 0.03090 | 0.03084 | 17.04 | 0.11 |
| 0.02026 | 0.02229 | 19.39 | 0.03467 | 0.03083 | 17.03 | 1.14 |
| 0.01533 | 0.01730 | 15.05 | 0.03360 | 0.03070 | 16.96 | 0.89 |
| 0.00143 | 0.00324 | 2.82 | 0.03092 | 0.03065 | 16.93 | 0.17 |
| -0.00069 | 0.00110 | 0.95 | 0.03052 | 0.03065 | 16.93 | 0.06 |
| 0.00034 | 0.00214 | 1.86 | 0.03067 | 0.03060 | 16.91 | 0.11 |
| -0.00002 | 0.00178 | 1.55 | 0.03060 | 0.03060 | 16.91 | 0.09 |
| 0.00092 | 0.00272 | 2.37 | 0.03077 | 0.03060 | 16.90 | 0.14 |
| -0.00071 | 0.00107 | 0.93 | 0.03038 | 0.03051 | 16.86 | 0.06 |
| -0.00090 | 0.00088 | 0.76 | 0.03031 | 0.03048 | 16.84 | 0.05 |
| -0.00040 | 0.00138 | 1.20 | 0.03035 | 0.03042 | 16.81 | 0.07 |
| 0.00042 | 0.00221 | 1.92 | 0.03050 | 0.03042 | 16.81 | 0.11 |
| -0.00011 | 0.00167 | 1.45 | 0.03040 | 0.03042 | 16.81 | 0.09 |
| 0.00178 | 0.00358 | 3.11 | 0.03074 | 0.03040 | 16.80 | 0.19 |
| -0.00041 | 0.00136 | 1.18 | 0.03028 | 0.03036 | 16.77 | 0.07 |
| 0.00060 | 0.00238 | 2.07 | 0.03043 | 0.03032 | 16.75 | 0.12 |
| 0.00000 | 0.00177 | 1.54 | 0.03028 | 0.03028 | 16.73 | 0.09 |
| 0.00287 | 0.00468 | 4.07 | 0.03082 | 0.03027 | 16.73 | 0.24 |
| -0.00071 | 0.00105 | 0.91 | 0.03012 | 0.03025 | 16.72 | 0.05 |
| 0.00383 | 0.00564 | 4.90 | 0.03095 | 0.03023 | 16.70 | 0.29 |
| 0.00181 | 0.00360 | 3.13 | 0.03049 | 0.03015 | 16.66 | 0.19 |
| -0.00136 | 0.00039 | 0.34 | 0.02987 | 0.03012 | 16.64 | 0.02 |
| -0.00079 | 0.00096 | 0.84 | 0.02997 | 0.03012 | 16.64 | 0.05 |
| 0.00398 | 0.00578 | 5.03 | 0.03084 | 0.03009 | 16.62 | 0.30 |
| -0.00051 | 0.00125 | 1.08 | 0.02993 | 0.03002 | 16.59 | 0.07 |
| 0.00203 | 0.00381 | 3.31 | 0.03037 | 0.02998 | 16.56 | 0.20 |
| -0.00041 | 0.00134 | 1.16 | 0.02989 | 0.02997 | 16.56 | 0.07 |
| 0.00170 | 0.00347 | 3.02 | 0.03028 | 0.02996 | 16.55 | 0.18 |
| -0.00066 | 0.00109 | 0.95 | 0.02982 | 0.02994 | 16.54 | 0.06 |
| -0.00090 | 0.00084 | 0.73 | 0.02974 | 0.02991 | 16.52 | 0.04 |
| -0.00093 | 0.00081 | 0.71 | 0.02971 | 0.02988 | 16.51 | 0.04 |
| 0.01456 | 0.01647 | 14.32 | 0.03263 | 0.02988 | 16.51 | 0.87 |
| 0.00145 | 0.00322 | 2.80 | 0.03014 | 0.02987 | 16.50 | 0.17 |
| 0.00084 | 0.00260 | 2.26 | 0.03002 | 0.02986 | 16.50 | 0.14 |
| 0.00215 | 0.00392 | 3.41 | 0.03026 | 0.02985 | 16.49 | 0.21 |
| 0.01581 | 0.01773 | 15.42 | 0.03283 | 0.02983 | 16.48 | 0.94 |
| 0.02775 | 0.02980 | 25.91 | 0.03507 | 0.02982 | 16.47 | 1.57 |
| 0.00040 | 0.00215 | 1.87 | 0.02987 | 0.02979 | 16.46 | 0.11 |
| -0.00008 | 0.00166 | 1.45 | 0.02975 | 0.02976 | 16.44 | 0.09 |
| -0.00091 | 0.00082 | 0.71 | 0.02959 | 0.02976 | 16.44 | 0.04 |
| 0.00212 | 0.00389 | 3.38 | 0.03016 | 0.02976 | 16.44 | 0.21 |
| 0.00095 | 0.00271 | 2.35 | 0.02993 | 0.02975 | 16.44 | 0.14 |
| 0.00166 | 0.00342 | 2.97 | 0.03005 | 0.02973 | 16.43 | 0.18 |
| 0.00242 | 0.00419 | 3.64 | 0.03017 | 0.02971 | 16.41 | 0.22 |
| 0.00100 | 0.00275 | 2.39 | 0.02987 | 0.02968 | 16.40 | 0.15 |
| -0.00074 | 0.00099 | 0.86 | 0.02952 | 0.02966 | 16.38 | 0.05 |
| 0.00072 | 0.00247 | 2.15 | 0.02979 | 0.02965 | 16.38 | 0.13 |
| 0.00177 | 0.00353 | 3.07 | 0.02998 | 0.02964 | 16.38 | 0.19 |
| 0.00040 | 0.00213 | 1.86 | 0.02966 | 0.02958 | 16.34 | 0.11 |
| -0.00243 | -0.00072 | -0.63 | 0.02911 | 0.02957 | 16.34 | -0.04 |
| 0.00131 | 0.00306 | 2.66 | 0.02981 | 0.02956 | 16.33 | 0.16 |
| 0.00169 | 0.00344 | 2.99 | 0.02984 | 0.02952 | 16.31 | 0.18 |
| 0.00159 | 0.00333 | 2.90 | 0.02980 | 0.02950 | 16.30 | 0.18 |
| -0.00083 | 0.00089 | 0.77 | 0.02933 | 0.02948 | 16.29 | 0.05 |
| 0.00153 | 0.00327 | 2.85 | 0.02975 | 0.02946 | 16.27 | 0.17 |
| -0.00104 | 0.00067 | 0.58 | 0.02915 | 0.02935 | 16.21 | 0.04 |
| 0.00304 | 0.00479 | 4.17 | 0.02990 | 0.02932 | 16.20 | 0.26 |
| -0.00079 | 0.00092 | 0.80 | 0.02912 | 0.02927 | 16.17 | 0.05 |
| 0.00130 | 0.00303 | 2.63 | 0.02949 | 0.02925 | 16.16 | 0.16 |
| 0.00104 | 0.00276 | 2.40 | 0.02942 | 0.02922 | 16.15 | 0.15 |
| 0.00121 | 0.00294 | 2.56 | 0.02944 | 0.02921 | 16.14 | 0.16 |
| -0.00074 | 0.00097 | 0.84 | 0.02906 | 0.02920 | 16.13 | 0.05 |
| 0.00417 | 0.00592 | 5.15 | 0.02996 | 0.02917 | 16.12 | 0.32 |
| -0.00070 | 0.00100 | 0.87 | 0.02896 | 0.02909 | 16.07 | 0.05 |
| 0.00117 | 0.00288 | 2.51 | 0.02931 | 0.02909 | 16.07 | 0.16 |
| -0.00067 | 0.00103 | 0.90 | 0.02895 | 0.02908 | 16.07 | 0.06 |
| 0.00091 | 0.00263 | 2.28 | 0.02924 | 0.02907 | 16.06 | 0.14 |
| -0.00099 | 0.00070 | 0.61 | 0.02887 | 0.02906 | 16.06 | 0.04 |
| -0.00067 | 0.00102 | 0.89 | 0.02886 | 0.02898 | 16.01 | 0.06 |
| 0.00048 | 0.00219 | 1.90 | 0.02906 | 0.02897 | 16.01 | 0.12 |
| 0.00238 | 0.00411 | 3.57 | 0.02942 | 0.02897 | 16.01 | 0.22 |
| 0.00309 | 0.00482 | 4.19 | 0.02954 | 0.02896 | 16.00 | 0.26 |
| 0.00211 | 0.00383 | 3.33 | 0.02935 | 0.02895 | 15.99 | 0.21 |
| -0.00095 | 0.00073 | 0.64 | 0.02876 | 0.02894 | 15.99 | 0.04 |
| -0.00063 | 0.00105 | 0.92 | 0.02880 | 0.02892 | 15.98 | 0.06 |
| 0.00090 | 0.00260 | 2.26 | 0.02909 | 0.02892 | 15.98 | 0.14 |
| 0.00209 | 0.00380 | 3.31 | 0.02931 | 0.02891 | 15.97 | 0.21 |
| 0.00046 | 0.00216 | 1.88 | 0.02898 | 0.02890 | 15.97 | 0.12 |
| -0.00100 | 0.00068 | 0.59 | 0.02867 | 0.02886 | 15.95 | 0.04 |
| -0.00079 | 0.00090 | 0.78 | 0.02871 | 0.02886 | 15.95 | 0.05 |
| 0.00112 | 0.00282 | 2.45 | 0.02901 | 0.02880 | 15.91 | 0.15 |
| -0.00093 | 0.00075 | 0.65 | 0.02858 | 0.02876 | 15.89 | 0.04 |
| 0.00185 | 0.00355 | 3.09 | 0.02907 | 0.02872 | 15.87 | 0.19 |
| -0.00053 | 0.00114 | 0.99 | 0.02859 | 0.02869 | 15.85 | 0.06 |
| -0.00079 | 0.00089 | 0.77 | 0.02852 | 0.02867 | 15.84 | 0.05 |
| 0.00170 | 0.00340 | 2.95 | 0.02896 | 0.02864 | 15.82 | 0.19 |
| -0.00060 | 0.00108 | 0.94 | 0.02852 | 0.02864 | 15.82 | 0.06 |
| 0.00156 | 0.00325 | 2.82 | 0.02883 | 0.02854 | 15.77 | 0.18 |
| 0.00117 | 0.00285 | 2.48 | 0.02875 | 0.02853 | 15.76 | 0.16 |
| 0.00048 | 0.00216 | 1.88 | 0.02857 | 0.02848 | 15.73 | 0.12 |
| 0.00165 | 0.00334 | 2.90 | 0.02878 | 0.02847 | 15.73 | 0.18 |
| 0.00111 | 0.00279 | 2.43 | 0.02863 | 0.02842 | 15.70 | 0.15 |
| -0.00088 | 0.00077 | 0.67 | 0.02822 | 0.02839 | 15.68 | 0.04 |
| 0.00130 | 0.00298 | 2.59 | 0.02862 | 0.02837 | 15.68 | 0.17 |
| -0.00112 | 0.00053 | 0.46 | 0.02816 | 0.02837 | 15.67 | 0.03 |
| 0.00035 | 0.00201 | 1.75 | 0.02842 | 0.02835 | 15.67 | 0.11 |
| 0.00192 | 0.00360 | 3.13 | 0.02871 | 0.02835 | 15.66 | 0.20 |
| -0.00070 | 0.00095 | 0.83 | 0.02817 | 0.02831 | 15.64 | 0.05 |
| 0.01459 | 0.01640 | 14.26 | 0.03099 | 0.02823 | 15.60 | 0.91 |
| 0.00070 | 0.00236 | 2.05 | 0.02836 | 0.02822 | 15.59 | 0.13 |
| 0.00211 | 0.00379 | 3.29 | 0.02860 | 0.02820 | 15.58 | 0.21 |
| 0.00050 | 0.00216 | 1.88 | 0.02829 | 0.02820 | 15.58 | 0.12 |
| -0.00067 | 0.00098 | 0.85 | 0.02807 | 0.02820 | 15.58 | 0.05 |
| 0.00163 | 0.00330 | 2.87 | 0.02849 | 0.02818 | 15.57 | 0.18 |
| 0.00079 | 0.00245 | 2.13 | 0.02832 | 0.02817 | 15.56 | 0.14 |
| 0.00190 | 0.00357 | 3.10 | 0.02848 | 0.02812 | 15.53 | 0.20 |
| 0.00006 | 0.00170 | 1.48 | 0.02813 | 0.02812 | 15.53 | 0.10 |
| 0.00465 | 0.00635 | 5.52 | 0.02898 | 0.02810 | 15.52 | 0.36 |
| 0.00129 | 0.00294 | 2.56 | 0.02829 | 0.02805 | 15.50 | 0.17 |
| 0.00225 | 0.00392 | 3.41 | 0.02839 | 0.02796 | 15.45 | 0.22 |
| 0.00020 | 0.00183 | 1.59 | 0.02787 | 0.02783 | 15.37 | 0.10 |
| 0.00281 | 0.00447 | 3.89 | 0.02834 | 0.02781 | 15.36 | 0.25 |
| 0.00100 | 0.00264 | 2.30 | 0.02798 | 0.02779 | 15.36 | 0.15 |
| -0.00039 | 0.00123 | 1.07 | 0.02767 | 0.02775 | 15.33 | 0.07 |
| -0.00081 | 0.00081 | 0.70 | 0.02756 | 0.02772 | 15.31 | 0.05 |
| -0.00040 | 0.00121 | 1.05 | 0.02761 | 0.02769 | 15.30 | 0.07 |
| 0.01923 | 0.02106 | 18.31 | 0.03126 | 0.02762 | 15.26 | 1.20 |
| 0.02296 | 0.02483 | 21.59 | 0.03196 | 0.02761 | 15.26 | 1.42 |
| 0.00027 | 0.00189 | 1.64 | 0.02763 | 0.02758 | 15.24 | 0.11 |
| 0.00025 | 0.00187 | 1.63 | 0.02763 | 0.02758 | 15.24 | 0.11 |
| -0.00048 | 0.00113 | 0.98 | 0.02746 | 0.02755 | 15.22 | 0.06 |
| -0.00052 | 0.00109 | 0.95 | 0.02743 | 0.02753 | 15.21 | 0.06 |
| 0.00021 | 0.00183 | 1.59 | 0.02756 | 0.02752 | 15.21 | 0.10 |
| 0.00034 | 0.00196 | 1.70 | 0.02758 | 0.02751 | 15.20 | 0.11 |
| 0.00084 | 0.00246 | 2.14 | 0.02767 | 0.02751 | 15.20 | 0.14 |
| -0.00116 | 0.00044 | 0.38 | 0.02725 | 0.02747 | 15.18 | 0.03 |
| 0.00188 | 0.00351 | 3.05 | 0.02780 | 0.02745 | 15.16 | 0.20 |
| -0.00087 | 0.00072 | 0.63 | 0.02724 | 0.02740 | 15.14 | 0.04 |
| 0.00160 | 0.00322 | 2.80 | 0.02762 | 0.02732 | 15.09 | 0.19 |
| 0.00211 | 0.00373 | 3.25 | 0.02771 | 0.02731 | 15.09 | 0.22 |
| 0.00054 | 0.00214 | 1.86 | 0.02737 | 0.02727 | 15.07 | 0.12 |
| 0.00044 | 0.00203 | 1.77 | 0.02721 | 0.02712 | 14.99 | 0.12 |
| -0.00096 | 0.00062 | 0.54 | 0.02692 | 0.02710 | 14.97 | 0.04 |
| 0.00062 | 0.00221 | 1.92 | 0.02720 | 0.02708 | 14.96 | 0.13 |
| -0.00072 | 0.00085 | 0.74 | 0.02690 | 0.02703 | 14.94 | 0.05 |
| 0.00044 | 0.00203 | 1.77 | 0.02710 | 0.02701 | 14.92 | 0.12 |
| -0.00012 | 0.00146 | 1.27 | 0.02690 | 0.02693 | 14.88 | 0.09 |
| -0.00088 | 0.00069 | 0.60 | 0.02675 | 0.02692 | 14.87 | 0.04 |
| 0.02252 | 0.02434 | 21.17 | 0.03117 | 0.02690 | 14.86 | 1.42 |
| -0.00014 | 0.00143 | 1.24 | 0.02685 | 0.02688 | 14.85 | 0.08 |
| -0.00061 | 0.00095 | 0.83 | 0.02671 | 0.02683 | 14.82 | 0.06 |
| 0.00004 | 0.00161 | 1.40 | 0.02675 | 0.02675 | 14.78 | 0.09 |
| 0.00061 | 0.00218 | 1.90 | 0.02682 | 0.02670 | 14.75 | 0.13 |
| 0.01261 | 0.01431 | 12.45 | 0.02906 | 0.02667 | 14.73 | 0.84 |
| 0.00271 | 0.00430 | 3.74 | 0.02717 | 0.02666 | 14.73 | 0.25 |
| 0.00055 | 0.00212 | 1.84 | 0.02676 | 0.02666 | 14.73 | 0.12 |
| 0.00039 | 0.00195 | 1.70 | 0.02671 | 0.02664 | 14.72 | 0.12 |
| -0.00033 | 0.00123 | 1.07 | 0.02656 | 0.02662 | 14.71 | 0.07 |
| -0.00077 | 0.00078 | 0.68 | 0.02646 | 0.02661 | 14.70 | 0.05 |
| 0.00108 | 0.00265 | 2.30 | 0.02679 | 0.02658 | 14.69 | 0.16 |
| 0.00394 | 0.00554 | 4.82 | 0.02731 | 0.02656 | 14.68 | 0.33 |
| -0.00001 | 0.00155 | 1.35 | 0.02653 | 0.02653 | 14.66 | 0.09 |
| -0.00052 | 0.00103 | 0.90 | 0.02643 | 0.02653 | 14.66 | 0.06 |
| 0.00087 | 0.00243 | 2.12 | 0.02663 | 0.02646 | 14.62 | 0.14 |
| -0.00094 | 0.00060 | 0.52 | 0.02628 | 0.02646 | 14.62 | 0.04 |
| 0.00201 | 0.00358 | 3.11 | 0.02678 | 0.02640 | 14.58 | 0.21 |
| -0.00025 | 0.00129 | 1.12 | 0.02634 | 0.02639 | 14.58 | 0.08 |
| 0.00078 | 0.00232 | 2.02 | 0.02640 | 0.02625 | 14.50 | 0.14 |
| -0.00122 | 0.00030 | 0.26 | 0.02601 | 0.02624 | 14.50 | 0.02 |
| 0.00076 | 0.00231 | 2.01 | 0.02635 | 0.02620 | 14.48 | 0.14 |
| 0.00066 | 0.00220 | 1.91 | 0.02631 | 0.02618 | 14.47 | 0.13 |
| 0.00102 | 0.00256 | 2.23 | 0.02636 | 0.02616 | 14.46 | 0.15 |
| 0.00071 | 0.00225 | 1.95 | 0.02627 | 0.02614 | 14.44 | 0.14 |
| 0.00155 | 0.00310 | 2.69 | 0.02641 | 0.02612 | 14.43 | 0.19 |
| 0.00111 | 0.00265 | 2.30 | 0.02625 | 0.02604 | 14.39 | 0.16 |
| 0.00054 | 0.00207 | 1.80 | 0.02613 | 0.02603 | 14.38 | 0.13 |
| -0.00033 | 0.00119 | 1.03 | 0.02597 | 0.02603 | 14.38 | 0.07 |
| 0.00008 | 0.00158 | 1.38 | 0.02567 | 0.02566 | 14.18 | 0.10 |
| 0.00016 | 0.00166 | 1.45 | 0.02566 | 0.02563 | 14.16 | 0.10 |
| 0.00062 | 0.00213 | 1.85 | 0.02571 | 0.02559 | 14.14 | 0.13 |
| 0.00211 | 0.00363 | 3.16 | 0.02598 | 0.02558 | 14.13 | 0.22 |
| 0.00051 | 0.00201 | 1.75 | 0.02567 | 0.02557 | 14.13 | 0.12 |
| 0.01094 | 0.01255 | 10.92 | 0.02762 | 0.02555 | 14.12 | 0.77 |
| 0.00067 | 0.00217 | 1.89 | 0.02567 | 0.02554 | 14.11 | 0.13 |
| -0.00055 | 0.00094 | 0.82 | 0.02544 | 0.02554 | 14.11 | 0.06 |
| 0.00116 | 0.00266 | 2.32 | 0.02566 | 0.02544 | 14.05 | 0.16 |
| 0.00015 | 0.00164 | 1.43 | 0.02544 | 0.02541 | 14.04 | 0.10 |
| 0.00013 | 0.00162 | 1.41 | 0.02529 | 0.02526 | 13.96 | 0.10 |
| 0.00044 | 0.00193 | 1.68 | 0.02533 | 0.02524 | 13.95 | 0.12 |
| -0.00051 | 0.00096 | 0.84 | 0.02511 | 0.02521 | 13.93 | 0.06 |
| 0.00040 | 0.00188 | 1.64 | 0.02525 | 0.02518 | 13.91 | 0.12 |
| 0.00013 | 0.00161 | 1.40 | 0.02515 | 0.02513 | 13.88 | 0.10 |
| 0.00040 | 0.00186 | 1.62 | 0.02505 | 0.02497 | 13.80 | 0.12 |
| 0.00025 | 0.00171 | 1.49 | 0.02502 | 0.02497 | 13.80 | 0.11 |
| 0.00192 | 0.00340 | 2.96 | 0.02532 | 0.02495 | 13.79 | 0.21 |
| 0.00298 | 0.00447 | 3.89 | 0.02548 | 0.02491 | 13.76 | 0.28 |
| 0.00219 | 0.00367 | 3.19 | 0.02530 | 0.02489 | 13.75 | 0.23 |
| -0.00125 | 0.00019 | 0.16 | 0.02458 | 0.02482 | 13.71 | 0.01 |
| 0.00016 | 0.00161 | 1.40 | 0.02482 | 0.02479 | 13.69 | 0.10 |
| 0.02325 | 0.02496 | 21.71 | 0.02916 | 0.02476 | 13.68 | 1.59 |
| -0.00004 | 0.00141 | 1.23 | 0.02473 | 0.02474 | 13.67 | 0.09 |
| -0.00094 | 0.00049 | 0.43 | 0.02452 | 0.02469 | 13.64 | 0.03 |
| 0.00129 | 0.00275 | 2.39 | 0.02493 | 0.02469 | 13.64 | 0.18 |
| 0.00375 | 0.00524 | 4.55 | 0.02529 | 0.02458 | 13.58 | 0.34 |
| 0.00027 | 0.00171 | 1.49 | 0.02462 | 0.02457 | 13.57 | 0.11 |
| -0.00058 | 0.00085 | 0.74 | 0.02437 | 0.02447 | 13.52 | 0.05 |
| 0.01141 | 0.01297 | 11.28 | 0.02663 | 0.02447 | 13.52 | 0.83 |
| -0.00094 | 0.00047 | 0.41 | 0.02419 | 0.02437 | 13.46 | 0.03 |
| 0.00035 | 0.00178 | 1.55 | 0.02442 | 0.02435 | 13.46 | 0.12 |
| -0.00029 | 0.00113 | 0.98 | 0.02425 | 0.02431 | 13.43 | 0.07 |
| 0.00420 | 0.00566 | 4.92 | 0.02500 | 0.02421 | 13.37 | 0.37 |
| 0.00057 | 0.00199 | 1.73 | 0.02421 | 0.02410 | 13.31 | 0.13 |
| 0.00890 | 0.01040 | 9.05 | 0.02570 | 0.02401 | 13.27 | 0.68 |
| 0.00155 | 0.00297 | 2.58 | 0.02427 | 0.02398 | 13.25 | 0.19 |
| 0.00310 | 0.00453 | 3.94 | 0.02452 | 0.02393 | 13.22 | 0.30 |
| 0.00260 | 0.00403 | 3.51 | 0.02440 | 0.02390 | 13.21 | 0.27 |
| 0.00515 | 0.00660 | 5.74 | 0.02471 | 0.02374 | 13.12 | 0.44 |
| -0.00028 | 0.00111 | 0.96 | 0.02367 | 0.02372 | 13.10 | 0.07 |
| 0.00145 | 0.00286 | 2.49 | 0.02399 | 0.02372 | 13.10 | 0.19 |
| 0.00302 | 0.00444 | 3.86 | 0.02425 | 0.02367 | 13.08 | 0.30 |
| 0.00137 | 0.00277 | 2.41 | 0.02383 | 0.02357 | 13.02 | 0.18 |
| -0.00025 | 0.00113 | 0.98 | 0.02346 | 0.02351 | 12.99 | 0.08 |
| 0.00136 | 0.00274 | 2.38 | 0.02363 | 0.02338 | 12.92 | 0.18 |
| -0.00025 | 0.00112 | 0.97 | 0.02333 | 0.02337 | 12.91 | 0.08 |
| -0.00037 | 0.00099 | 0.86 | 0.02311 | 0.02318 | 12.81 | 0.07 |
| 0.00194 | 0.00332 | 2.88 | 0.02342 | 0.02305 | 12.74 | 0.23 |
| 0.00006 | 0.00140 | 1.22 | 0.02302 | 0.02301 | 12.71 | 0.10 |
| -0.00023 | 0.00111 | 0.97 | 0.02294 | 0.02298 | 12.70 | 0.08 |
| 0.00363 | 0.00501 | 4.36 | 0.02363 | 0.02295 | 12.68 | 0.34 |
| -0.00148 | -0.00016 | -0.14 | 0.02252 | 0.02280 | 12.60 | -0.01 |
| 0.00157 | 0.00292 | 2.54 | 0.02308 | 0.02278 | 12.59 | 0.20 |
| 0.00022 | 0.00155 | 1.34 | 0.02258 | 0.02254 | 12.45 | 0.11 |
| -0.00168 | -0.00039 | -0.34 | 0.02213 | 0.02245 | 12.40 | -0.03 |
| -0.00056 | 0.00074 | 0.65 | 0.02231 | 0.02242 | 12.38 | 0.05 |
| -0.00011 | 0.00119 | 1.04 | 0.02226 | 0.02228 | 12.31 | 0.08 |
| -0.00017 | 0.00112 | 0.98 | 0.02202 | 0.02205 | 12.18 | 0.08 |
| -0.00100 | 0.00028 | 0.24 | 0.02185 | 0.02204 | 12.18 | 0.02 |
| 0.00154 | 0.00284 | 2.47 | 0.02212 | 0.02183 | 12.06 | 0.20 |
| -0.00059 | 0.00068 | 0.60 | 0.02171 | 0.02182 | 12.05 | 0.05 |
| -0.00056 | 0.00071 | 0.62 | 0.02163 | 0.02174 | 12.01 | 0.05 |
| 0.00249 | 0.00379 | 3.30 | 0.02217 | 0.02169 | 11.99 | 0.28 |
| 0.01690 | 0.01836 | 15.97 | 0.02487 | 0.02167 | 11.97 | 1.33 |
| -0.00059 | 0.00068 | 0.59 | 0.02155 | 0.02166 | 11.97 | 0.05 |
| 0.00438 | 0.00570 | 4.95 | 0.02245 | 0.02162 | 11.95 | 0.41 |
| 0.00283 | 0.00413 | 3.59 | 0.02209 | 0.02155 | 11.91 | 0.30 |
| -0.00063 | 0.00063 | 0.55 | 0.02142 | 0.02154 | 11.90 | 0.05 |
| 0.00301 | 0.00430 | 3.74 | 0.02197 | 0.02140 | 11.82 | 0.32 |
| -0.00094 | 0.00029 | 0.25 | 0.02105 | 0.02123 | 11.73 | 0.02 |
| 0.00357 | 0.00485 | 4.22 | 0.02186 | 0.02118 | 11.70 | 0.36 |
| 0.00233 | 0.00360 | 3.13 | 0.02160 | 0.02116 | 11.69 | 0.27 |
| 0.00177 | 0.00303 | 2.63 | 0.02149 | 0.02116 | 11.69 | 0.23 |
| 0.00083 | 0.00208 | 1.81 | 0.02131 | 0.02115 | 11.69 | 0.15 |
| -0.00009 | 0.00115 | 1.00 | 0.02110 | 0.02112 | 11.67 | 0.09 |
| 0.00025 | 0.00148 | 1.29 | 0.02111 | 0.02106 | 11.64 | 0.11 |
| 0.00411 | 0.00539 | 4.69 | 0.02181 | 0.02103 | 11.62 | 0.40 |
| 0.00075 | 0.00199 | 1.73 | 0.02102 | 0.02088 | 11.54 | 0.15 |
| 0.00002 | 0.00123 | 1.07 | 0.02062 | 0.02061 | 11.39 | 0.09 |
| 0.00281 | 0.00404 | 3.51 | 0.02098 | 0.02044 | 11.30 | 0.31 |
| 0.00418 | 0.00543 | 4.72 | 0.02122 | 0.02043 | 11.29 | 0.42 |
| 0.00217 | 0.00338 | 2.94 | 0.02075 | 0.02034 | 11.24 | 0.26 |
| -0.00002 | 0.00117 | 1.02 | 0.02033 | 0.02033 | 11.23 | 0.09 |
| 0.00648 | 0.00774 | 6.73 | 0.02149 | 0.02027 | 11.20 | 0.60 |
| 0.00195 | 0.00316 | 2.75 | 0.02063 | 0.02026 | 11.19 | 0.25 |
| 0.00261 | 0.00383 | 3.33 | 0.02073 | 0.02024 | 11.18 | 0.30 |
| 0.00891 | 0.01020 | 8.87 | 0.02190 | 0.02022 | 11.17 | 0.79 |
| -0.00025 | 0.00093 | 0.81 | 0.02013 | 0.02018 | 11.15 | 0.07 |
| -0.00025 | 0.00093 | 0.80 | 0.01998 | 0.02003 | 11.07 | 0.07 |
| 0.00169 | 0.00288 | 2.51 | 0.02033 | 0.02001 | 11.06 | 0.23 |
| 0.00075 | 0.00193 | 1.68 | 0.02015 | 0.02001 | 11.06 | 0.15 |
| 0.00189 | 0.00308 | 2.68 | 0.02033 | 0.01997 | 11.03 | 0.24 |
| 0.00122 | 0.00240 | 2.09 | 0.02011 | 0.01988 | 10.98 | 0.19 |
| -0.00036 | 0.00080 | 0.70 | 0.01979 | 0.01985 | 10.97 | 0.06 |
| 0.01912 | 0.02049 | 17.82 | 0.02337 | 0.01975 | 10.91 | 1.63 |
| 0.00033 | 0.00149 | 1.30 | 0.01979 | 0.01972 | 10.90 | 0.12 |
| 0.00124 | 0.00241 | 2.09 | 0.01994 | 0.01970 | 10.89 | 0.19 |
| -0.00002 | 0.00113 | 0.99 | 0.01963 | 0.01963 | 10.85 | 0.09 |
| 0.00053 | 0.00168 | 1.46 | 0.01964 | 0.01954 | 10.80 | 0.14 |
| -0.00017 | 0.00098 | 0.85 | 0.01951 | 0.01954 | 10.80 | 0.08 |
| 0.00119 | 0.00235 | 2.04 | 0.01976 | 0.01954 | 10.79 | 0.19 |
| -0.00053 | 0.00060 | 0.52 | 0.01937 | 0.01947 | 10.76 | 0.05 |
| 0.00081 | 0.00196 | 1.70 | 0.01956 | 0.01941 | 10.72 | 0.16 |
| 0.00155 | 0.00270 | 2.35 | 0.01966 | 0.01937 | 10.70 | 0.22 |
| 0.00086 | 0.00200 | 1.74 | 0.01951 | 0.01935 | 10.69 | 0.16 |
| 0.00267 | 0.00383 | 3.33 | 0.01983 | 0.01933 | 10.68 | 0.31 |
| 0.00407 | 0.00524 | 4.56 | 0.02002 | 0.01925 | 10.64 | 0.43 |
| 0.02935 | 0.03080 | 26.78 | 0.02470 | 0.01914 | 10.58 | 2.53 |
| 0.00005 | 0.00117 | 1.02 | 0.01915 | 0.01914 | 10.58 | 0.10 |
| -0.00002 | 0.00110 | 0.96 | 0.01911 | 0.01911 | 10.56 | 0.09 |
| 0.00022 | 0.00133 | 1.16 | 0.01894 | 0.01889 | 10.44 | 0.11 |
| 0.00491 | 0.00607 | 5.28 | 0.01974 | 0.01881 | 10.39 | 0.51 |
| 0.00213 | 0.00325 | 2.83 | 0.01909 | 0.01868 | 10.32 | 0.27 |
| 0.00221 | 0.00332 | 2.88 | 0.01894 | 0.01853 | 10.24 | 0.28 |
| 0.00456 | 0.00569 | 4.95 | 0.01938 | 0.01852 | 10.23 | 0.48 |
| 0.00243 | 0.00352 | 3.07 | 0.01871 | 0.01825 | 10.08 | 0.30 |
| 0.00483 | 0.00595 | 5.17 | 0.01914 | 0.01823 | 10.07 | 0.51 |
| 0.00702 | 0.00817 | 7.10 | 0.01952 | 0.01819 | 10.05 | 0.71 |
| 0.00156 | 0.00263 | 2.29 | 0.01837 | 0.01808 | 9.99 | 0.23 |
| 0.00130 | 0.00237 | 2.06 | 0.01823 | 0.01798 | 9.94 | 0.21 |
| 0.00126 | 0.00232 | 2.02 | 0.01805 | 0.01781 | 9.84 | 0.20 |
| 0.00012 | 0.00115 | 1.00 | 0.01765 | 0.01763 | 9.74 | 0.10 |
| 0.00089 | 0.00192 | 1.67 | 0.01766 | 0.01749 | 9.66 | 0.17 |
| 0.00002 | 0.00105 | 0.91 | 0.01746 | 0.01746 | 9.64 | 0.09 |
| 0.02083 | 0.02208 | 19.20 | 0.02137 | 0.01742 | 9.63 | 1.99 |
| -0.00071 | 0.00030 | 0.26 | 0.01722 | 0.01736 | 9.59 | 0.03 |
| 0.00454 | 0.00560 | 4.87 | 0.01812 | 0.01726 | 9.54 | 0.51 |
| 0.00498 | 0.00604 | 5.25 | 0.01810 | 0.01716 | 9.48 | 0.55 |
| 0.02515 | 0.02643 | 22.99 | 0.02191 | 0.01715 | 9.48 | 2.43 |
| 0.00202 | 0.00305 | 2.65 | 0.01748 | 0.01709 | 9.44 | 0.28 |
| 0.00287 | 0.00391 | 3.40 | 0.01762 | 0.01708 | 9.43 | 0.36 |
| 0.00024 | 0.00124 | 1.08 | 0.01711 | 0.01707 | 9.43 | 0.11 |
| 0.01516 | 0.01632 | 14.19 | 0.01989 | 0.01702 | 9.40 | 1.51 |
| -0.00029 | 0.00071 | 0.61 | 0.01694 | 0.01700 | 9.39 | 0.07 |
| 0.00075 | 0.00174 | 1.52 | 0.01702 | 0.01688 | 9.33 | 0.16 |
| 0.00267 | 0.00369 | 3.20 | 0.01738 | 0.01688 | 9.32 | 0.34 |
| 0.00113 | 0.00213 | 1.85 | 0.01706 | 0.01685 | 9.31 | 0.20 |
| 0.00245 | 0.00345 | 3.00 | 0.01706 | 0.01659 | 9.17 | 0.33 |
| 0.00087 | 0.00185 | 1.61 | 0.01663 | 0.01646 | 9.09 | 0.18 |
| 0.00029 | 0.00124 | 1.08 | 0.01637 | 0.01631 | 9.01 | 0.12 |
| 0.00003 | 0.00098 | 0.86 | 0.01625 | 0.01625 | 8.98 | 0.10 |
| 0.00168 | 0.00265 | 2.31 | 0.01656 | 0.01624 | 8.97 | 0.26 |
| 0.00002 | 0.00096 | 0.84 | 0.01613 | 0.01613 | 8.91 | 0.09 |
| 0.00406 | 0.00505 | 4.39 | 0.01685 | 0.01608 | 8.88 | 0.49 |
| 0.00291 | 0.00388 | 3.38 | 0.01658 | 0.01603 | 8.86 | 0.38 |
| 0.00025 | 0.00119 | 1.03 | 0.01590 | 0.01585 | 8.76 | 0.12 |
| 0.00387 | 0.00483 | 4.20 | 0.01649 | 0.01576 | 8.71 | 0.48 |
| 0.00371 | 0.00468 | 4.07 | 0.01644 | 0.01574 | 8.70 | 0.47 |
| 0.00006 | 0.00098 | 0.85 | 0.01574 | 0.01573 | 8.69 | 0.10 |
| 0.00069 | 0.00162 | 1.41 | 0.01585 | 0.01572 | 8.68 | 0.16 |
| 0.00018 | 0.00111 | 0.96 | 0.01575 | 0.01571 | 8.68 | 0.11 |
| 0.00529 | 0.00627 | 5.45 | 0.01665 | 0.01565 | 8.65 | 0.63 |
| 0.00046 | 0.00138 | 1.20 | 0.01569 | 0.01560 | 8.62 | 0.14 |
| 0.00068 | 0.00160 | 1.39 | 0.01568 | 0.01555 | 8.59 | 0.16 |
| 0.00300 | 0.00394 | 3.43 | 0.01609 | 0.01552 | 8.57 | 0.40 |
| 0.00028 | 0.00119 | 1.03 | 0.01554 | 0.01549 | 8.56 | 0.12 |
| 0.00590 | 0.00687 | 5.97 | 0.01648 | 0.01537 | 8.49 | 0.70 |
| 0.00255 | 0.00346 | 3.01 | 0.01565 | 0.01517 | 8.38 | 0.36 |
| 0.00242 | 0.00333 | 2.89 | 0.01550 | 0.01504 | 8.31 | 0.35 |
| 0.00159 | 0.00248 | 2.16 | 0.01531 | 0.01501 | 8.29 | 0.26 |
| 0.00660 | 0.00753 | 6.55 | 0.01598 | 0.01473 | 8.14 | 0.80 |
| 0.00288 | 0.00376 | 3.27 | 0.01505 | 0.01450 | 8.01 | 0.41 |
| 0.00229 | 0.00317 | 2.75 | 0.01487 | 0.01444 | 7.98 | 0.35 |
| 0.00173 | 0.00259 | 2.25 | 0.01460 | 0.01427 | 7.88 | 0.29 |
| 0.00130 | 0.00215 | 1.87 | 0.01441 | 0.01417 | 7.83 | 0.24 |
| 0.00060 | 0.00144 | 1.25 | 0.01425 | 0.01414 | 7.81 | 0.16 |
| 0.00763 | 0.00853 | 7.42 | 0.01537 | 0.01393 | 7.70 | 0.96 |
| 0.01265 | 0.01360 | 11.83 | 0.01623 | 0.01384 | 7.64 | 1.55 |
| 0.00102 | 0.00182 | 1.58 | 0.01375 | 0.01355 | 7.49 | 0.21 |
| 0.00342 | 0.00425 | 3.70 | 0.01414 | 0.01350 | 7.46 | 0.50 |
| 0.00548 | 0.00632 | 5.49 | 0.01432 | 0.01328 | 7.34 | 0.75 |
| -0.00025 | 0.00052 | 0.45 | 0.01313 | 0.01318 | 7.28 | 0.06 |
| 0.00426 | 0.00508 | 4.41 | 0.01391 | 0.01311 | 7.24 | 0.61 |
| 0.00333 | 0.00414 | 3.60 | 0.01370 | 0.01307 | 7.22 | 0.50 |
| 0.00024 | 0.00100 | 0.87 | 0.01304 | 0.01299 | 7.18 | 0.12 |
| 0.00033 | 0.00109 | 0.94 | 0.01298 | 0.01292 | 7.14 | 0.13 |
| 0.00509 | 0.00590 | 5.13 | 0.01382 | 0.01285 | 7.10 | 0.72 |
| 0.00353 | 0.00432 | 3.76 | 0.01348 | 0.01281 | 7.08 | 0.53 |
| 0.00202 | 0.00278 | 2.42 | 0.01309 | 0.01271 | 7.02 | 0.34 |
| -0.00005 | 0.00068 | 0.59 | 0.01239 | 0.01240 | 6.85 | 0.09 |
| 0.00070 | 0.00143 | 1.24 | 0.01249 | 0.01236 | 6.83 | 0.18 |
| 0.00011 | 0.00083 | 0.72 | 0.01228 | 0.01226 | 6.77 | 0.11 |
| 0.00687 | 0.00766 | 6.66 | 0.01353 | 0.01223 | 6.76 | 0.99 |
| 0.00520 | 0.00596 | 5.19 | 0.01307 | 0.01209 | 6.68 | 0.78 |
| 0.00283 | 0.00356 | 3.09 | 0.01232 | 0.01178 | 6.51 | 0.47 |
| 0.00426 | 0.00500 | 4.34 | 0.01252 | 0.01172 | 6.47 | 0.67 |
| 0.00556 | 0.00631 | 5.49 | 0.01271 | 0.01165 | 6.44 | 0.85 |
| 0.00425 | 0.00497 | 4.33 | 0.01242 | 0.01162 | 6.42 | 0.67 |
| 0.00334 | 0.00406 | 3.53 | 0.01225 | 0.01161 | 6.42 | 0.55 |
| 0.00041 | 0.00109 | 0.94 | 0.01149 | 0.01141 | 6.31 | 0.15 |
| -0.00047 | 0.00019 | 0.17 | 0.01125 | 0.01134 | 6.27 | 0.03 |
| -0.00025 | 0.00041 | 0.36 | 0.01119 | 0.01124 | 6.21 | 0.06 |
| 0.00174 | 0.00241 | 2.10 | 0.01150 | 0.01117 | 6.17 | 0.34 |
| 0.00190 | 0.00256 | 2.23 | 0.01137 | 0.01101 | 6.08 | 0.37 |
| 0.00492 | 0.00562 | 4.89 | 0.01190 | 0.01097 | 6.06 | 0.81 |
| 0.00208 | 0.00274 | 2.39 | 0.01134 | 0.01095 | 6.05 | 0.39 |
| 0.00576 | 0.00646 | 5.62 | 0.01196 | 0.01087 | 6.01 | 0.94 |
| 0.00084 | 0.00149 | 1.29 | 0.01101 | 0.01085 | 5.99 | 0.22 |
| 0.00603 | 0.00673 | 5.85 | 0.01184 | 0.01070 | 5.91 | 0.99 |
| 0.00340 | 0.00406 | 3.53 | 0.01119 | 0.01055 | 5.83 | 0.61 |
| 0.00468 | 0.00530 | 4.61 | 0.01061 | 0.00972 | 5.37 | 0.86 |
| 0.00240 | 0.00299 | 2.60 | 0.01007 | 0.00962 | 5.31 | 0.49 |
| 0.00727 | 0.00791 | 6.87 | 0.01084 | 0.00947 | 5.23 | 1.31 |
| -0.00060 | -0.00007 | -0.06 | 0.00909 | 0.00920 | 5.08 | -0.01 |
| 0.00192 | 0.00247 | 2.15 | 0.00945 | 0.00909 | 5.02 | 0.43 |
| 0.00430 | 0.00487 | 4.23 | 0.00962 | 0.00880 | 4.86 | 0.87 |
| 0.00797 | 0.00856 | 7.45 | 0.01015 | 0.00864 | 4.77 | 1.56 |
| 0.00024 | 0.00072 | 0.62 | 0.00815 | 0.00811 | 4.48 | 0.14 |
| 0.00357 | 0.00405 | 3.52 | 0.00814 | 0.00747 | 4.13 | 0.85 |
| -0.00049 | -0.00006 | -0.06 | 0.00730 | 0.00739 | 4.09 | -0.01 |
| -0.00012 | 0.00016 | 0.14 | 0.00479 | 0.00481 | 2.66 | 0.05 |
| -0.00019 | 0.00006 | 0.05 | 0.00421 | 0.00425 | 2.35 | 0.02 |
| -0.00005 | 0.00019 | 0.17 | 0.00409 | 0.00410 | 2.26 | 0.07 |
| -0.00048 | -0.00026 | -0.23 | 0.00376 | 0.00385 | 2.13 | -0.11 |
| -0.00007 | 0.00014 | 0.12 | 0.00357 | 0.00358 | 1.98 | 0.06 |
| -0.00078 | -0.00058 | -0.50 | 0.00341 | 0.00356 | 1.97 | -0.26 |
| -0.00094 | -0.00075 | -0.66 | 0.00313 | 0.00330 | 1.83 | -0.36 |
| 0.00005 | 0.00023 | 0.20 | 0.00312 | 0.00311 | 1.72 | 0.12 |
| -0.00069 | -0.00055 | -0.48 | 0.00243 | 0.00256 | 1.41 | -0.34 |
| -0.00087 | -0.00075 | -0.65 | 0.00210 | 0.00227 | 1.25 | -0.52 |
| -0.00004 | 0.00009 | 0.07 | 0.00214 | 0.00215 | 1.19 | 0.06 |
| -0.00031 | -0.00020 | -0.17 | 0.00185 | 0.00191 | 1.05 | -0.17 |
| 0.00006 | 0.00016 | 0.14 | 0.00164 | 0.00163 | 0.90 | 0.15 |
| 0.00302 | 0.00312 | 2.71 | 0.00171 | 0.00114 | 0.63 | 4.29 |
| -0.00083 | -0.00077 | -0.67 | 0.00094 | 0.00109 | 0.60 | -1.11 |
| 0.00248 | 0.00256 | 2.23 | 0.00136 | 0.00089 | 0.49 | 4.55 |
| -0.00013 | -0.00008 | -0.07 | 0.00086 | 0.00088 | 0.49 | -0.15 |
| 0.00038 | 0.00043 | 0.37 | 0.00082 | 0.00075 | 0.41 | 0.91 |
| 0.00653 | 0.00663 | 5.77 | 0.00174 | 0.00050 | 0.28 | 20.81 |
| 0.01522 | 0.01542 | 13.41 | 0.00332 | 0.00044 | 0.24 | 55.67 |
| -0.00090 | -0.00089 | -0.78 | 0.00006 | 0.00023 | 0.12 | -6.24 |
| -0.00031 | -0.00030 | -0.26 | 0.00012 | 0.00018 | 0.10 | -2.68 |
| -0.00051 | -0.00050 | -0.44 | 0.00008 | 0.00018 | 0.10 | -4.51 |
| -0.00021 | -0.00021 | -0.18 | 0.00012 | 0.00016 | 0.09 | -2.04 |
| -0.00017 | -0.00016 | -0.14 | 0.00012 | 0.00015 | 0.08 | -1.67 |
| 0.00019 | 0.00020 | 0.17 | 0.00012 | 0.00008 | 0.05 | 3.74 |

^a^ activity ratio was the activity of PAAS/mutant to that of ECAP in their fused form.
